# Enhanced oxygen evolution over dual corner-shared cobalt tetrahedra

**DOI:** 10.1038/s41467-022-33000-w

**Published:** 2022-09-20

**Authors:** Yubo Chen, Joon Kyo Seo, Yuanmiao Sun, Thomas A. Wynn, Marco Olguin, Minghao Zhang, Jingxian Wang, Shibo Xi, Yonghua Du, Kaidi Yuan, Wei Chen, Adrian C. Fisher, Maoyu Wang, Zhenxing Feng, Jose Gracia, Li Huang, Shixuan Du, Hong-Jun Gao, Ying Shirley Meng, Zhichuan J. Xu

**Affiliations:** 1 School of Material Science and Engineering, Nanyang Technological University, 50 Nanyang Avenue, Singapore, 639798 Singapore; 2 The Cambridge Centre for Advanced Research and Education in Singapore, 1 CREATE way, Singapore, 138602 Singapore; 3 Solar Fuels Laboratory, Nanyang Technological University, 50 Nanyang Avenue, Singapore, 639798 Singapore; 4 Energy Research Institute @ Nanyang Technological University, 50 Nanyang Avenue, Singapore, 639798 Singapore; 5 Department of Nano Engineering, University of California San Diego, 9500 Gilman Drive, La Jolla, CA 92093 USA; 6 Materials Science and Engineering, University of California San Diego, 9500 Gilman Drive, La Jolla, CA 92093 USA; 7 Gwangju Clean Energy Research Center, Korea Institute of Energy Research, Gwangju, 61003 Republic of Korea; 8 Institute of Chemical and Engineering Sciences, A*STAR, 1 Pesek Road, Singapore, 627833 Singapore; 9 Department of Physics, National University of Singapore, 2 Science Drive 3, Singapore, 117542 Singapore; 10 Department of Chemical Engineering, University of Cambridge, Cambridge, CB2 3RA UK; 11 School of Chemical, Biological, and Environmental Engineering, Oregon State University, Corvallis, OR 97331 USA; 12 MagnetoCat SL, General Polavieja 9 3I, Alicante, 03012 Spain; 13 Beijing National Laboratory for Condensed Matter Physics and Institute of Physics, Chinese Academy of Science, Beijing, 100190 China; 14 Pritzker School of Molecular Engineering, University of Chicago, Chicago, IL 60637 USA

**Keywords:** Electrocatalysis, Electrocatalysis, Materials for energy and catalysis, Renewable energy

## Abstract

Developing efficient catalysts is of paramount importance to oxygen evolution, a sluggish anodic reaction that provides essential electrons and protons for various electrochemical processes, such as hydrogen generation. Here, we report that the oxygen evolution reaction (OER) can be efficiently catalyzed by cobalt tetrahedra, which are stabilized over the surface of a Swedenborgite-type YBCo_4_O_7_ material. We reveal that the surface of YBaCo_4_O_7_ possesses strong resilience towards structural amorphization during OER, which originates from its distinctive structural evolution toward electrochemical oxidation. The bulk of YBaCo_4_O_7_ composes of corner-sharing only CoO_4_ tetrahedra, which can flexibly alter their positions to accommodate the insertion of interstitial oxygen ions and mediate the stress during the electrochemical oxidation. The density functional theory calculations demonstrate that the OER is efficiently catalyzed by a binuclear active site of dual corner-shared cobalt tetrahedra, which have a coordination number switching between 3 and 4 during the reaction. We expect that the reported active structural motif of dual corner-shared cobalt tetrahedra in this study could enable further development of compounds for catalyzing the OER.

## Abstract (web summary)

Efficient oxygen evolution relies on the development of promising catalysts. Herein, the authors demonstrate that cobalt tetrahedra, stabilized over the surface of YBCo_4_O_7_ material, can catalyze oxygen evolution reaction efficiently.

## Introduction

A sustainable society relies upon renewable energy resources instead of fossil fuel combustion to generate electricity, reducing emissions of pollutants. Meanwhile, several eco-friendly energy conversion systems, such as water electrolyzers and metal-air batteries, have been explored to store energy from renewable resources. In these systems, the oxygen evolution reaction (OER) is an essential step. The complex four-electron transfer process makes OER occurs at a large overpotential and thus generally consumes considerable energy. In the last years, various transition-metal-based complex compounds have been intensively explored for catalyzing the OER efficiently^1–10^. However, the design of advanced catalysts with high activity and sustainability is still challenging owing to limited choices^11,12^.

The OER active site in octahedral geometry (or square pyramidal geometry for an undercoordinated surface site) has been dominant and is generally regarded as a promising local structure with a coordination number switching between 5 and 6 during the OER^13–15^. Active transition-metal with lower coordination numbers (in trigonal bipyramidal geometry) has also been reported with a coordination number switching between 4 and 5 during the OER^16,17^. These active sites in a reduced coordinate environment possess unique electronic and structural characteristics, which are found beneficial for the charge-transfer and adsorption of intermediates during the electrochemical reaction ^16–18^.

Till now, little attention has been paid to the possible active sites of tri-/tetra-oxygen-coordinated (in tetrahedral geometry), which have the potential to enable efficient OER. Compared with the octahedral moieties, the tetrahedral moieties are more flexible, and the tetrahedral units are easier of deformation and rotation, which can be beneficial for the formation of surface-active sites for a promoted OER^19,20^. A few complex materials, such as spinel, wurtzite, and phosphates, containing tetrahedral sites, have been considered for catalyzing OER^16,21–23^. These tetrahedral sites, however, are generally found inactive and are prone to transform to actual catalytic sites with higher coordination numbers (5 or 6)^8,16,21–23^. Nevertheless, the formation of a stable tri-oxygen-coordinated site on the surface from the material containing tetrahedral moieties is possible. For instance, an active tri-oxygen-coordinated aluminum site has been observed from the surface of gamma alumina, which contains tetrahedra and has been extensively used as the catalyst or catalyst support in the petroleum industry ^24,25^.

In this work, we demonstrate that tri-oxygen-coordinated cobalt can be stabilized over the surface of a YBaCo_4_O_7_ (YBC4) material, which is composed of corner-shared CoO_4_ tetrahedra^26^. An active motif of dual corner-shared cobalt tetrahedra can efficiently catalyze the OER as well as the cobalt in an octahedral geometry.

## Results and discussion

### Cobalt tetrahedra over YBC4 surface

**Fig. 1:**
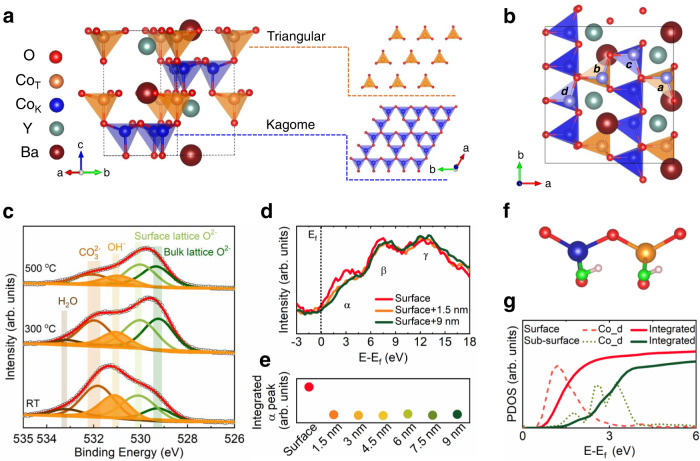
Cobalt tetrahedra over YBC4 surface. **a** Crystal structure of YBC4 and the arrangements of corner-shared CoO4 tetrahedra in the Triangular layer and the Kagome layer. **b** The top view of the YBaCo_4_O_7_ (110) facet with tri-oxygen-coordinated cobalt (marked in orchid). **c** O 1 s core spectra from temperature-dependent XPS of an as-prepared YBC4 sample. **d** O K-edge EELS spectra from the sub-surface (~9 nm and ~1.5 nm) and the surface of YBC4. All spectra are aligned to Fermi energy (*E*_f_) with 0 eV. **e** Extracted intensities of *α* peaks from sub-surface to surface. A linear background is applied to subtract the background adsorption. **f** A typical OH-adsorbed site, including adjacent Co from the Kagome layer and the Triangular layer, over the (110) facet. The O from adsorbed OH group is highlighted in green. **g** PDOS of unoccupied Co *3d* states from sub-surface (dot line) and surface (dash line). The solid lines are the corresponding integrated PDOS of unoccupied Co *3d* states.

The corner-shared CoO_4_ tetrahedra in YBC4 are packed along the c-direction (Fig. 1a) with alternated Triangular and Kagome layers. The Y and Ba cations occupy octahedral and anticuboctahedral cavities within the CoO_4_ tetrahedra, respectively. An averaged valence state of +2.25 for Co is expected in YBaCo_4_O_7_. A sol-gel method was applied to synthesize the YBaCo_4_O_7_. The as-prepared YBaCo_4_O_7_ shows a high phase purity with dominant corner-shared CoO_4_ tetrahedra, as confirmed by the synchrotron-based X-ray diffraction (XRD) refinement, high-resolution transmission electron microscopy (HR-TEM), and X-ray absorption near edge structure (XANES) analysis (Figs. S1–3 and Table S1). As the OER occurs over the surface of a catalyst, the surface structure of the as-prepared YBaCo_4_O_7_ is comprehensively studied. From the calculated energies of typical facets and the Wulff constructions for YBC4, the YBC4 polycrystalline is found dominant with the (110) surface (Fig. S4), as shown in Fig.1b. On such a computational (110) surface, the cleavage of one of the oxygen atoms from the Co-sites is predicted, and the formed tri-oxygen-coordinated Co locates in either the Triangular layer (*a&b*) or Kagome layer (*c&d*). Additionally, it can be found that the local environment of *a&c* sites and *b&d* sites resemble each other. The rationality and stability of such tri-oxygen-coordinated Co are further supported by the similar crystal orbital overlap populations (COOPs) of Co-O bonds from surface tri-oxygen-coordinated Co, surface tetra-oxygen-coordinated Co, and bulk tetra-oxygen-coordinated Co (Fig. S5). Note that the highly active tri-oxygen-coordinated Al site, observed from the surface of alumina, can be further stabilized by hydroxylation^25^. Regarding the surface of YBC4 in ambient air and solution, the exposed tri-oxygen-coordinated Co should also bond with the OH group from the external environment. This hypothesis is demonstrated through the high-temperature X-ray photoelectron spectroscopy (HT-XPS) in vacuum (Fig. 1c, Fig. S6, 7 and Table S2). As shown in Fig. 1c, five characteristic peaks from bulk lattice oxygen, surface lattice oxygen, OH group, carbonate, and water molecular, can be identified^27–31^. The results in Fig. S6 suggest that the OH group is adsorbed on the surface cobalt. Due to the thermal-induced desorption, the intensities of the peaks from the OH group, carbonate, and water molecular gradually decrease or disappear when heating the sample to 500 °C. The above results indicate that the heat treatment can produce the tri-coordinated Co with the desorption of OH groups over the surface of YBC4. To resolve the structure of the OH-adsorbed Co over (110) surface, the most likely active site toward catalyzing the OER, combined scanning transmission electron microscope-electron energy loss spectroscopy (STEM-EELS) and density functional theory (DFT) calculations are performed. Along the [110] direction, EELS spectra were collected from the sub-surface to the surface (Fig. S8). As is demonstrated by the measured energy difference between the Co-L_3_ peak and the Co-L_2_ peak (Fig. S9, 10), the cobalt over the surface and the sub-surface regions are dominant with a divalent state. The O K-edge spectra, originating from excitations of the O 1s shell to unoccupied *2p* states, are shown in Fig. S11, and three representative spectra are shown in Fig. 1d. Three characteristic peaks can be observed from the spectrum, labeled as *α*, *β*, and γ, respectively. The *α* peak corresponds to the excitation from the O 1s state to the hybridized O *2p*-Co *3d* state based on the calculated projected-density-of-state (PDOS) from YBC4 bulk (Fig. S12). A similar α peak is also observed in the previously measured O K-edge from YBC4 using X-ray absorption spectroscopy^32^. The *β* and the γ peaks are attributed to the excitation from the O 1s state to the hybridized O *2p*-(Y *4d*/Ba *5d*) state and O *2p*-Co *4sp* state, respectively. In the spectrum from the outer surface, the *β* and γ peaks resemble the ones from the sub-surface. The overlapped *β* peaks indicate that the state of hybridization between O *2p* and Y *4d*/Ba *5d* is similar at the sub-surface and the surface. However, as compared with the spectra from the sub-surface, the *α* peak in the spectrum from the surface shows an apparent left shift with increased intensity (Fig. 1e), indicating the electronic structure of surface Co is different from the one in the lattice CoO_4_ tetrahedron. Specifically, the surface Co can also be tetra-oxygen-coordinated, but by bonding with an additional OH group. This deduction is further demonstrated by the DFT calculations. Figure 1f shows a typical OH-adsorbed site, including representative adjacent Co (*b&d*) from either the Kagome layer or Triangular layer, over the (110) surface. The corresponding PDOS of Co from this surface site and another adjacent CoO_4_ tetrahedra (Kagome layer and Triangular layer) in the sub-surface are calculated (Fig. S13). The unoccupied Co *3d* states from the surface and the sub-surface are integrated (Fig. 1g). Compared with the sub-surface Co, the PDOS of unoccupied surface Co *3d* states shows an apparent shift towards the Fermi level. Moreover, the PDOS of unoccupied Co *3d* states at the surface shows an increased integral area, contributing to the increased intensity of *α* peak from the surface O K-edge spectra.

### OER activity and stability

**Fig. 2:**
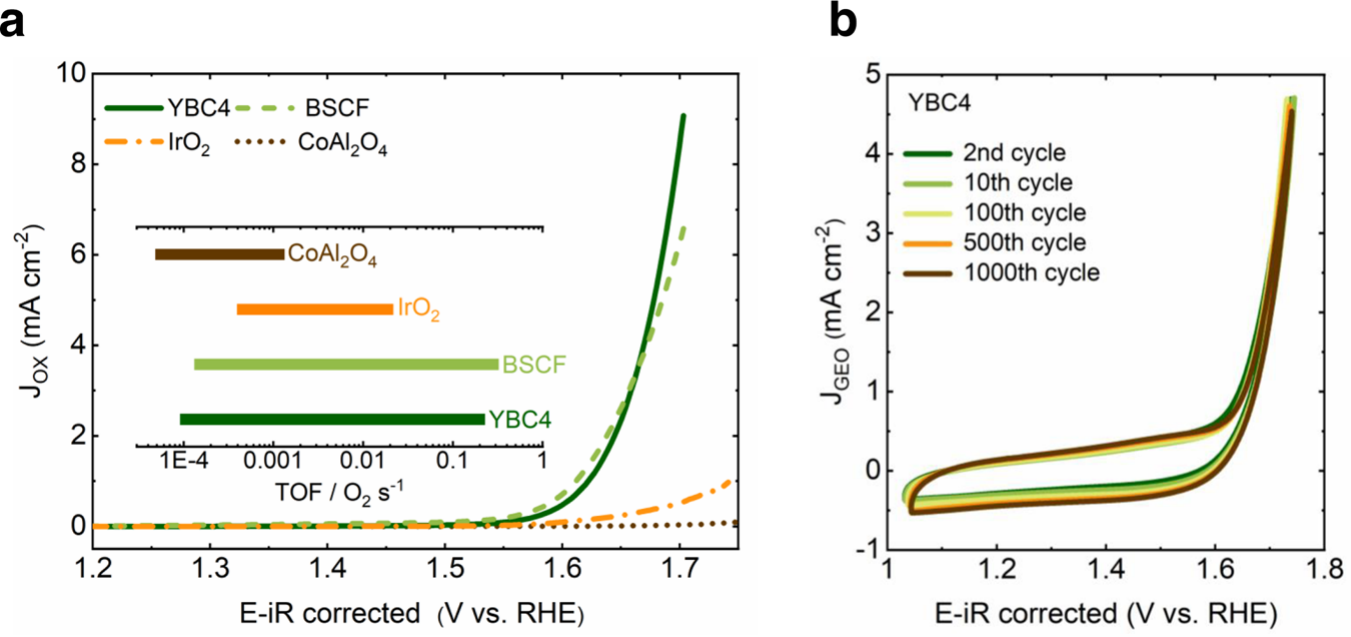
OER activity and stability. **a** iR-corrected and oxides surface area normalized OER currents at a scan rate of 10 mV/s. The inset is the turnover frequency (TOF) for YBC4, BSCF, CoAl_2_O_4_, and IrO_2_ at an overpotential of 300 mV. **b** A consecutive CV scanning test for YBC4.

The OER catalytic activity of YBC4 was measured using the rotating-disk electrode (RDE) technique in 0.1 M KOH (pH = 12.82). The result is compared with spinel-type CoAl_2_O_4_, perovskite-type BSCF, and rutile-type IrO_2_. The high phase purity of as-prepared CoAl_2_O_4_ and BSCF is confirmed by XRD (Fig. S14). The iR-corrected and oxide surface area (Fig. S15, evaluated by BET method) normalized cyclic voltammetry (CV) curves at a scan rate of 10 mV/s are presented in Fig. S16. The capacitance correction is further applied for the second CV cycle and Fig. 2a presents the OER currents of YBC4, BSCF, IrO_2_, and CoAl_2_O_4._ A Faradaic efficiency of 96.5 ± 1.4% is measured for YBC4 (Fig. S17), confirming that the measured current mainly comes from oxygen evolution. The catalytic activity of YBC4 outperforms those of IrO_2_ and CoAl_2_O_4_. A comparable activity is observed between the YBC4 and the BSCF. The turnover frequency (TOF), including lower limits and upper limits, is calculated at an overpotential of 300 mV (the inset in Fig. 2a). The cobalt in YBC4 shows an upper limit TOF of ~2.27 × 10^−1^ S^−1^, which is approximately 2 orders of magnitude higher than ~1.33 × 10^−3^ S^−1^ of CoAl_2_O_4_, approximately ten times higher than ~2.15 × 10^−2^ S^−1^ of IrO_2_ and comparable with the TOF of the BSCF (~3.22 × 10^−1^ S^−1^). This activity trend is further demonstrated with the corresponding Tafel plots (shown in Fig. S18). CV cycling was then performed to evaluate the stability of YBC4. As shown in Fig. 2b, in 1000 CV cycles (scanned for approximately 28 h at a scan rate of 20 mV/s), the CV curves from representative cycles highly overlap with each other, indicating a stable OER activity of YBC4. Note that, from the evolution of CV curves between ~1 V and ~1.65 V, slightly increased capacitance during cycling can be observed. Such capacitance increment is found related to the electrode substrate (graphite paper) and is different from the case in the BSCF electrode, which shows steeply increased capacitance due to the surface instability of BSCF (Fig. S19). The steady performance during CV cycling manifests a stable surface structure of the YBC4.

### Enhanced stability by the flexible structure

**Fig. 3:**
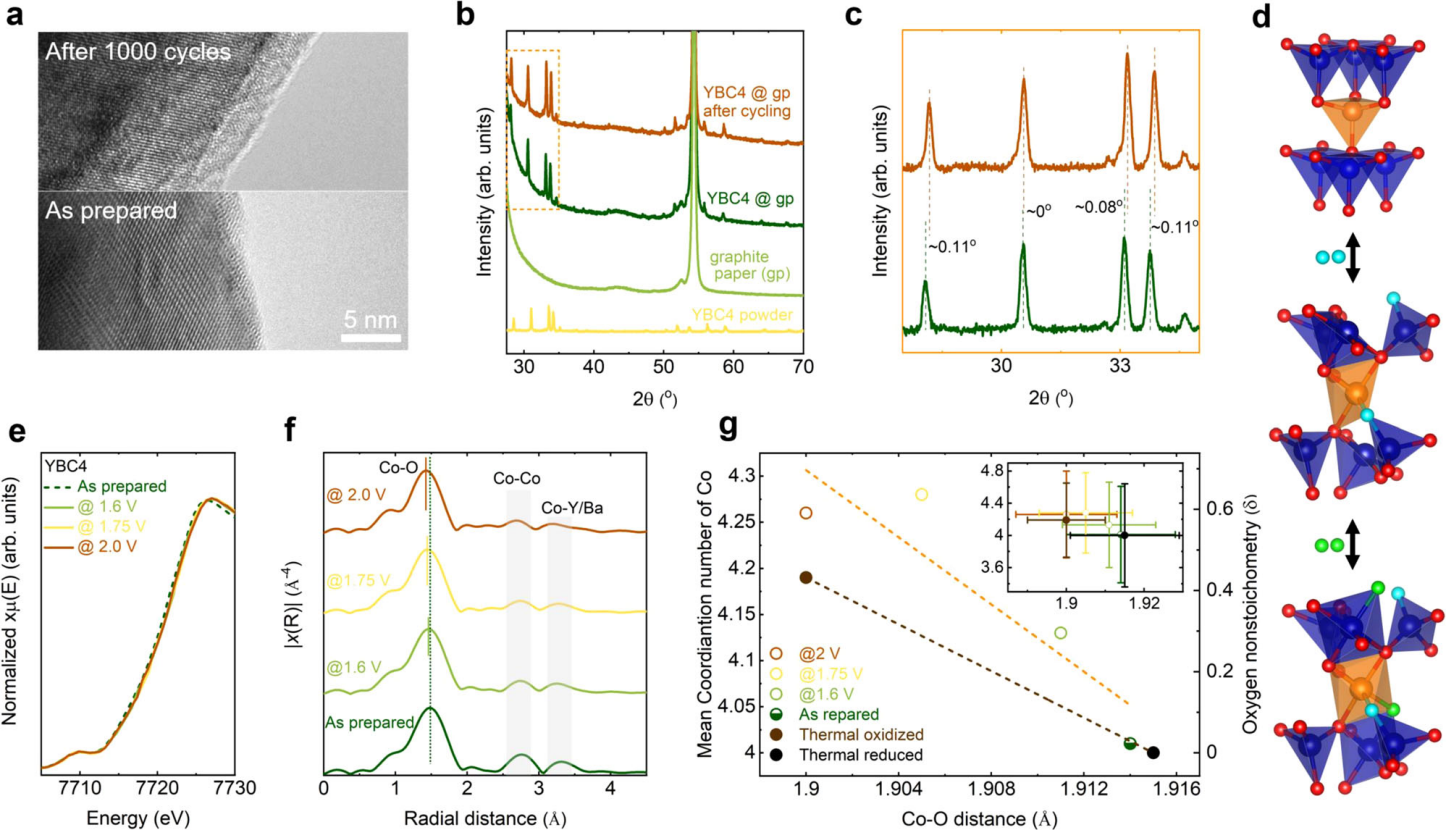
Enhanced stability by the flexible structure. **a** HRTEM images of surface structures from the as-prepared YBC4 and 1000-cycled YBC4. **b** The XRD patterns of the as-prepared YBC4 powder, electrode substrate of graphite paper (gp), and YBC4@gp before and after 1000 cycles of CV cycling. **c** The details of peak shifts in the XRD patterns of YBC4@gp before and after CV cycling. **d** An oxygen intercalation process in the YBC4 lattice. The sky-blue spheres and green spheres represent oxygen atoms inserted into the lattice. **e** Measured Co K-edge XANES of the as-prepared and electrochemically oxidized YBC4. **f** k^3^-weighted Co K-edge EXAFS spectra of the as-prepared and electrochemically oxidized YBC4. **g** The Co-O distance, the mean coordination number of Co, and oxygen nonstoichiometry (δ) in the as-prepared and the thermally/electrochemically treated YBC4. The inset shows the error bar (SD) for the data. The oxygen nonstoichiometry is estimated based on the mean coordination number from a YBaCo_4_O_7_ and YBaCo_4_O_8_^34^. The average coordination number in YBaCo_4_O_7_ and YBaCo_4_O_8_ is 4 and 4.44, respectively.

To pinpoint the root cause for the observed stability in YBC4, HR-TEM, XRD, ICP-MS, XPS, and XAS were conducted to characterize the bulk and surface before, during, and after the electrochemical tests. Figure 3a and Fig. S20 show the TEM images from the surfaces of YBC4 before and after CV cycling. Both the pristine and the cycled YBC4 show well-crystallized surfaces. Correspondingly, the dissolution of Y and Ba is also found negligible in the electrolyte used for the CV testing (Table S3). From the evolution of Co *2p* core spectra after CV cycling (Fig. S21 and Table S4), the YBC4 is electrochemically oxidized during OER. As reflected by the right shift of the peaks (Fig. 3b, c) in the XRD pattern of YBC4 after CV cycling, the YBC4 lattice contracts during OER. Note that such XRD peak-shift resembles the case from a thermally oxidized YBC4, indicating the similar structural evolution of YBC4 during the electrochemical and thermal oxidation (Fig. S22). A representative thermal oxidation process of YBC4 is presented in Fig. 3d, where the tetrahedra can rotate within the lattice during oxidation and partial cobalt atoms will coordinate with the intercalated oxygen^33,34^. The electrochemical oxidation in complex oxides can introduce the ionic size mismatch and strain within the lattice, leading to structural instability. For example, in  perovskite, a highly oxidized state with ionic size mismatch induces structural collapse and cation exclusion (See Fig. S23 for additional discussions)^35,36^. Similarly, surface structural amorphization and/or transformation are observed in typical catalysts, such as perovskite-type BSCF and spinel-type Co_3_O_4_, when a sufficient high overpotential (a highly oxidizing environment) is applied for catalyzing OER^21,22,37^. For a better understanding of the electrochemical oxidation of YBC4 during OER, we performed additional XAS analysis on three YBC4 samples, which had been electrochemically oxidized at 1.6, 1.75, and 2 V (vs. RHE without iR-correction) for 500 mins, respectively. From the XANES spectra of Co K-edge shown in Fig. 3e, a right shift can be observed from all three electrochemically oxidized samples, indicating the oxidation of Co during OER. Such oxidation induces unique structural evolution, which can be detected from the corresponding extended X-ray absorption fine structure (EXAFS) spectra in Fig. 3f. Three representative peaks, related to the first Co-O shell (~1.5 Å), closed pairs of Co-Co shell (~2.7 Å), and Co-Y/Ba shell (~3.2 Å), can be observed in the as-prepared YBC4. After electrochemical oxidation, the first peak gradually shifts to a lower distance as the applied potential increases, indicating a reduced Co-O distance. On the other hand, the intensity of the second and the third peaks drastically reduces after electrochemical oxidation, indicating a greatly increased distortion of CoO_4_ tetrahedra within the YBC4 lattice. The measured XRD patterns (Fig. S24) confirm the electrochemically oxidized samples are still highly crystallized, except for the peak shifts due to the lattice contraction. Moreover, the observed EXAFS changes due to electrochemical oxidation resemble the cases in thermally oxidized YBC4 (Fig. S25). Based on the EXAFS fitting (Table S5), the Co-O distance, mean coordination number of Co, and oxygen nonstoichiometry (δ, which represents the difference between oxygen stoichiometry and 7) in as-prepared, thermally treated, and electrochemically treated YBC4 are summarized in Fig. 3g. A similar trend in the contraction of Co-O bond (due to Co oxidation) and increment of Co coordination and oxygen nonstoichiometry are revealed. Note that even for the YBC4 oxidized at 2.0 V, the oxygen nonstoichiometry (~0.6) is still far below the upper limit of approximately 1.5 in YBC4^38^. Finally, the stress of lattice caused by cation oxidation and oxygen intercalation during OER can be accommodated by the unique local structural rearrangement in YBC4 (Fig. 3d). The extraction of large Y and Ba, which occurs in perovskites, is then alleviated in YBC4 during the OER tests.

### OER mechanism over cobalt tetrahedra

It has been proposed that the Co octahedra are responsible for the OER catalytic activity in a more oxidized YBaCo_4_O_7.3_ catalyst^39^. Because of the oxygen nonstoichiometry (*δ* ≈ 0.18), in as-prepared YBC4, a few Co atoms (~ 4%) are in an octahedral geometry. These Co octahedra, however, are found cannot be the key to the catalytic activity of as-prepared YBC4. This is because the catalytic activity of a stoichiometric YBC4 (*δ* ≈ 0) is as good as the as-prepared YBC4 (Fig. S26). Thus, the measured activity of as-prepared YBC4 originates from the dominant Co tetrahedra, or more specifically, surface tri-/tetra-oxygen-coordinated Co.

**Fig. 4:**
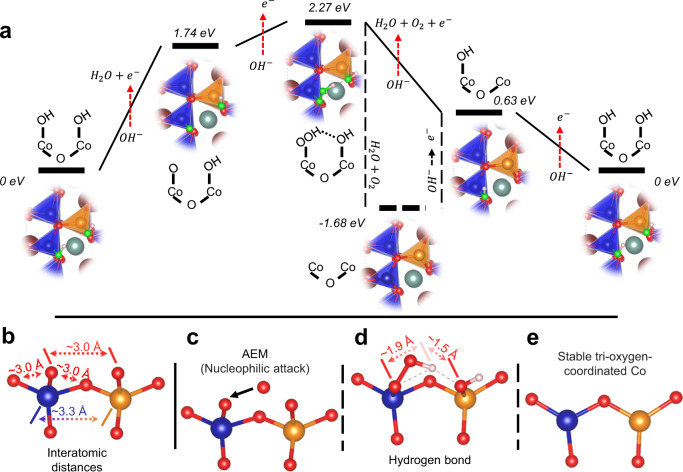
Hydrogen bond. **a** The calculated most likely paths in the OER on the YBC4 (110) surface. The free energies of different possible surface statuses in each proton-coupled electron transfer step are also presented. The O involved in the OER is highlighted in green. **b** The active structural motif of mono-µ-oxo-bridged Co in a tetrahedral geometry. **c**–**e** The key intermediate steps in the proposed OER mechanism.

To depict our hypothesis on how OER is catalyzed over the surface Co tetrahedra, DFT calculations were performed with a computational hydrogen electrode (CHE) mode to explore the possible interactions between surface tri-oxygen-coordinated Co and OER intermediates^40^. The possible OER paths over the Co tetrahedra are illustrated in Fig. S27. Figure 4a presents the free energy diagrams for the most likely OER process, and the detailed OER steps are expressed with the following equations:

1 $$\begin{array}{c}\left(\begin{array}{c}\triangle \\ k\end{array}*{{{{{\rm{OH}}}}}}\cdots \begin{array}{c}\triangle \\ t\end{array}*{{{{{\rm{OH}}}}}}\right)+{{{{{{\rm{OH}}}}}}}^{-}\to \left(\begin{array}{c}\triangle \\ k\end{array}*O\cdots \begin{array}{c}\triangle \\ t\end{array}*{{{{{\rm{OH}}}}}}\right)+{\rm {H}}_{2}{\rm {O}}+{{{{{{\rm{e}}}}}}}^{-}\end{array}$$

2 $$\begin{array}{c}\left(\begin{array}{c}\triangle \\ k\end{array}*{{{{{\rm{O}}}}}}\cdots \begin{array}{c}\triangle \\ t\end{array}*{{{{{\rm{OH}}}}}}\right)+{{{{{{\rm{OH}}}}}}}^{-}\to \left(\begin{array}{c}\triangle \\ k\end{array}*{\rm {OOH}}\cdots \begin{array}{c}\triangle \\ t\end{array}*{{{{{\rm{OH}}}}}}\right)+{{{{{{\rm{e}}}}}}}^{-}\end{array}$$

3 $$\begin{array}{c}\left(\begin{array}{c}\triangle \\ k\end{array}*{{{{{\rm{OOH}}}}}}\cdots \begin{array}{c}\triangle \\ t\end{array}*{{{{{\rm{OH}}}}}}\right)\to \left(\begin{array}{c}\triangle \\ k\end{array}*\cdots \begin{array}{c}\triangle \\ t\end{array}*\right)+{\rm {H}}_{2}{\rm {O}}+{\rm {O}}_{2}\end{array}$$

4 $$\begin{array}{c}\left(\begin{array}{c}\triangle \\ k\end{array}*\cdots \begin{array}{c}\triangle \\ t\end{array}*\right)+{{{{{{\rm{OH}}}}}}}^{-}\to \left(\begin{array}{c}\triangle \\ k\end{array}*{{{{{\rm{OH}}}}}}\cdots \begin{array}{c}\triangle \\ t\end{array}*\right)+{{{{{{\rm{e}}}}}}}^{-}\end{array}$$

5 $$\begin{array}{c}\left(\begin{array}{c}\triangle \\ k\end{array}*{{{{{\rm{OH}}}}}}\cdots \begin{array}{c}\triangle \\ t\end{array}*\right)+{{{{{{\rm{OH}}}}}}}^{-}\to \left(\begin{array}{c}\triangle \\ k\end{array}*{{{{{\rm{OH}}}}}}\cdots \begin{array}{c}\triangle \\ t\end{array}*{{{{{\rm{OH}}}}}}\right)+{{{{{{\rm{e}}}}}}}^{-}\end{array}$$

Where $\left({{\triangle} \atop {k}}*{{{{{\rm{OH}}}}}}\cdots {{\triangle} \atop {t}}*{{{{{\rm{OH}}}}}}\right)$ represents the initial active sites of adjacent OH-adsorbed Co tetrahedra. ${{\triangle} \atop {k}} \ast$ and ${{\triangle} \atop {t}} \ast$ correspond to the surface tri-oxygen-coordinated Co sites from the Kagome and the Triangular layer, respectively. In this mechanism, the OER starts from the adjacent Co sites with mono-µ-oxo-bridged CoO3(OH) in a tetrahedral geometry $\left({{\triangle} \atop {k}}*{{{{{\rm{OH}}}}}}\cdots {{\triangle} \atop {t}}*{{{{{\rm{OH}}}}}}\right)$. In the first proton-coupled electron transfer (PCET) step, the deprotonation occurs at the ${{\triangle} \atop {k}}*{{{{{\rm{OH}}}}}}$ site. Then, an OO bond is formed (${{\triangle} \atop {k}}*{{{{{\rm{OOH}}}}}}$) after the nucleophilic attack. And the $\left({{\triangle} \atop {k}}*{{{{{\rm{OOH}}}}}}\cdots {{\triangle} \atop {t}}*{{{{{\rm{OH}}}}}}\right)$ is stabilized by the strong hydrogen bonding interaction between ${{\triangle} \atop {k}}*{{{{{\rm{OOH}}}}}}$ and ${{\triangle} \atop {t}}*{{{{{\rm{OH}}}}}}$. Subsequently, the O_2_ releases from the ${{\triangle} \atop {k}}*{{{{{\rm{OOH}}}}}}$ coupled with the formation of adjacent tri-oxygen-coordinated Co (${{\triangle} \atop {k}}*\cdots {{\triangle} \atop {t}}*$). By bonding with another two OH groups through the PCET mechanism, the (${{\triangle} \atop {k}}*\cdots {{\triangle} \atop {t}}*$) resets to the initial status (${{\triangle} \atop {k}}*{{{{{\rm{OH}}}}}}\cdots {{\triangle} \atop {t}}*{{{{{\rm{OH}}}}}}$). Overall, this OER process shows a theoretical overpotential of 510 mV, which is 50 mV lower than a calculated overpotential of 560 mV from BSCF. Note that an experimental overpotential difference of 50 mV may indicate an OER current change by several times. However, a theoretical overpotential difference of 50 mV can be related to the error of DFT calculation^41^. Based on the proposed mechanism, the enhanced OER can be ascribed to the unique surface-active site of mono-µ-oxo-bridged Co tetrahedra, which origins from the arrangement of CoO_4_ tetrahedra in YBC4 (Fig. 1a and Fig. S28).

The particularity of mono-µ-oxo-bridged Co in tetrahedral geometry is further studied by comparing it with Co in octahedral geometry, which has been extensively explored for catalyzing OER. Through an in-depth comparison of two coordination geometries, a single CoO_4_ tetrahedron in YBC4 is found non-optimal toward catalyzing the OER, which unlike many state-of-the-art catalysts with the finely tuned octahedral active site (Supplementary Discussion, Table S6). The measured activity of mono-µ-oxo-bridged Co in tetrahedral geometry (dual corner-shared cobalt tetrahedra) is attributed to its unique geometry structure (Supplementary Discussion, Fig. S29, and S30), which enables the proposed OER mechanism. Specifically, Fig. 4b presents the mono-µ-oxo-bridged Co in tetrahedral geometry, and three typical interatomic distances of adjacent O in a single Co polyhedron, adjacent Co, and two terminal O from adjacent Co are marked. Firstly, it is found that the relatively large interatomic distances of adjacent and two terminal O atoms make the coupling of adjacent and two terminal O atoms unfavorable, and, as a result, a water nucleophilic attack induced O–O bond formation (adsorption evolution mechanism, AEM) is preferred (Fig. 4c). Secondly, the adjacent Co-Co distance is short enough to allow the interaction of intermediates from two Co centers, i.e., hydrogen bonding stabilized $\left({{\triangle} \atop {k}}*{{{{{\rm{OOH}}}}}}\cdots {{\triangle} \atop {t}}*{{{{{\rm{OH}}}}}}\right)$, which enables the bypass of scaling relation (Fig. 4d). The formation of such a hydrogen bond, however, is impossible in a single CoO_4_ unit. Additionally, given that the lattice-oxygen-mediated OER is also unlikely in YBC4 (Supplementary Discussion of Charge-transfer energy), a single CoO_4_ site would not enable the circumvented scaling relation. Thirdly, the stable tri-oxygen-coordinated Co site (${{\triangle} \atop {k}}*\cdots {{\triangle} \atop {t}}*$) over YBC4 (110) surface facilitates the coupled desorption of O_2_ (from ${{\triangle} \atop {k}}*{\rm {OO}}$) and H_2_O (from ${{\triangle} \atop {t}}*{\rm {O}}{\rm {H}}_{2}$) (Fig. 4e). Finally, most of these active mono-µ-oxo-bridged Co in tetrahedral geometry retained after the electrochemical oxidation-induced structure evaluation (Supplementary Discussion, Fig. S31).

In summary, we propose and demonstrate that a YBC4 material, with stabilized surface tri-oxygen-coordinated Co, exhibits an activity superior to that of IrO_2_ and comparable with that of the BSCF. The flexible bulk structure can accommodate the volume change induced by electrochemical oxidation and thus greatly enhance the resistance to surface reconstruction during OER. The active motif for OER is ascribed to the dual corner-shared cobalt tetrahedra. These discoveries will likely widen the perspective of electrocatalyst design and lead to the development of new types of oxide catalysts with symmetry-induced topological states through different coordination environments.

## Methods

### Preparation and property characterization of oxides

YBC4, BSCF, and CoAl_2_O_4_ oxides were synthesized with a sol-gel method. Shortly, proper mole ratios of raw metal nitrates were weighed and dissolved in deionized water from a Millipore Milli-Q system (resistivity equals to 18.2 MΩ). EDTA and citrate acid were then added into the solution at a molar ratio of 1: 1.5. The solution was stirred and heated to evaporate the water. Finally, a concentrated gel was formed, which was then heated at approximately 250 °C for 5 h to burn most of the organics. The solid precursor was further calcined in air at 1000 °C for 12 h (YBC4 and BSCF) and 800 °C for 5 h (CoAl_2_O_4_). All samples were furnace cool down to room temperature. The IrO_2_ (99.9% trace metals basis) was purchased from Sigma-Aldrich Co.

The phase structure of the YBC4 oxide was characterized with synchrotron-based XRD with a beamline 17-BM at the Advanced Photon Source (APS) of Argonne National Laboratory. The wavelength of the X-ray was 0.72768 Å. XRD measurements on BSCF and CoAl_2_O_4_ and catalysts loaded on electrodes were performed with a Bruker D8 Advance in the Bragg-Brentano geometry using a Copper Kα radiation. The structures were analyzed by the Rietveld method with the GSAS program and EXPGUI interface ^42^.

X-ray adsorption spectroscopy (XAS) experiments were performed at 9BM of Advanced Photon Source, Argonne National Laboratory and Singapore Synchrotron Light Source, XAFCA beamline. The energy axis was calibrated with Co foils between the transmission (*I*_t_) and reference (*I*_ref_) ion chambers. Data analyses were performed with the Athena software package. Specifically, for the modeling of extended X-ray absorption fine structure spectra, the range of the Fourier transform from k-space was 2.766-11.175 Å^−1^. The fitting range in R-range was from 1 Å to 2.2 Å, and the Hanning window was used. The fits were performed in R-space, and the fitting range in R-range was from 1 Å to 2.2 Å. The position of the edge E_0_ was defined as the first inflection point in the main rise of µ(E). The phase functions were generated with Feff6 in the Demeter package^43^. A standard YBaCo_4_O_7_ structure, with the space group of P *6*_*3*_ *mc*, was used as the initial model.

TEM images and EELS spectra were collected on JEM-2010F (JEOL) equipped with a Gatan 963 Quantum GIF SE. TEM image simulation was performed with JEMS software. The EELS spectra were acquired at a beam size of ~0.7 Å and the acquisition time of each spectrum was 100 s. The background of EELS spectra was subtracted based on a power-law model with GMS 3 software.

The room temperature XPS tests for the sample before and after electrochemical tests were performed on PHI5000 VersaProbe spectrometer including an Al Kα X-ray source. For the HT-XPS characterization, Omicron® Mg Kα source (hν = 1253.6 eV) and hemisphere analyzer were used with a fixed passing energy of 20 eV for each element. The system was calibrated to the Fermi level without additional adjustment as the samples had good electric conductivity. For the sample preparation, the YBC4 powder (approximately 1 g) was pressed into a pellet (10 mm in diameter). The sample pellet was then degassed in the ultrahigh vacuum (UHV) chamber where both the consequent heat-treatment and XPS measurements were carried out. All the data were fitted by the XPSPEAK software package. Linear-type background subtraction was applied, and the spectra were fitted using a combined Lorentzian-Gaussian line shape. The Co *2p* binding energy values reported by Gautiera et al. were used while fitting the Co *2p* spectrum ^44^.

The surface areas were characterized by nitrogen adsorption-desorption tests (ASAP Tri-star II 3020) with the Brunauer-Emmett-Teller (BET) method.

### Electrode preparation and electrochemical characterization

Glassy carbon (rotating disk) electrode with an area of 0.196 cm^−2^ was pre-polished with 50 nm Al_2_O_3_ powder and then sonicated in deionized water for at least three times to remove the residual Al_2_O_3_ particles. The catalyst ink with a concentration of 5 mg mL^−1^ was prepared by ultra-sonically dispersing 5 mg oxide catalyst and 2 mg acetylene black (Alfa Aesar) in a water (750 µL)-isopropanol (225 µL)-Nafion (25 µL) solution. 10 µL of well-dispersed ink was dropped onto the glassy carbon and dried overnight. The electrochemical experiments were carried out in a 150 mL glass cell at room temperature with an RDE system (Pine Instrument). An SP-150 workstation was applied to perform cyclic voltammetry scanning, chrono potentiometric tests, and electrochemical impedance spectroscopy analysis. A rotation speed of 1600 rpm was used. Before tests, the glassware was boiled in water to remove contaminants. The tests were performed in a 0.1 M KOH solution, which was purged with ultra-pure oxygen before each measurement for 30 min. A Pt wire was used as the counter electrode and a saturated calomel electrode (SCE) was used for reference. The evolution of oxygen and the Faradic efficiency were evaluated with rotating ring-disk electrode (RRDE) experiments. The loading of the YBC4 catalyst was identical to the case in RDE experiments. During the measurements, the potential of the Pt-ring disk was maintained at a constant potential of 0.3 V (vs RHE) and, to minimize the bubble formation, an RRDE rotation rate of 3000 rpm was used.

For CV cycling tests, a graphite paper (~2.5 cm×2.5 cm) was used as the support and the catalyst loading amount is 0.25 mg cm^−2^. The test was performed in a homemade electrochemical polyethylene cell with a Pt wire as the counter electrode and a mercuric oxide electrode as the reference electrode. The concentration of dissolved cations during electrochemical cycling in the electrolyte is measured with an inductively coupled plasma mass spectrometer (ICP-MS, Elan DRC-e). For the tests of electrochemical oxidation of YBC4 with chronoamperometry, a graphite paper (~1 cm × 2 cm) was used as the support and the loading amount of catalyst increased to 0.5 mg cm^−2^.

### Calculation of turnover frequency (TOF)

The TOF was calculated from the equation:

$${{{{{\rm{TOF}}}}}}=\frac{j\times {A}_{{{{{{{\rm{OX}}}}}}}}}{4\times e\times {N}_{A}}$$

where *j* is the BET surface area normalized current density at an overpotential of 300 mV. *A*_OX_ is the total surface area of the catalyst deposited on the GC electrode. *e* is the electric charge carried by a single electron. *N*_*A*_ is the number of active sites. While calculating the *N*_*A*_, we assumed that either all Co/Ir atoms in the catalysts are active (lower limits) or only the Co/Ir atoms on the surface are active (upper limits). Then, a range for the TOF of different catalysts is presented. Refined lattice parameters of different materials were applied to calculate the densities and the exposed number of surface-exposed Co/Ir atoms. To simplify the process, we assumed a (110) surface for YBC4, a (100) surface for BSCF^45^, a (100) surface for CoAl_2_O_4_^46^, and a (110) surface for IrO_2_^47^.

### Computation details

First-principles calculations were conducted based on the spin-polarized Generalized Gradient Approximation (GGA) using the Perdew-Burke-Ernzerhof (PBE) exchange and correlation functionals implemented in Density Functional Theory (DFT)^48–50^. We utilized a plane-wave basis set with a cutoff energy of 1.3 times the maximum cutoff specified by the pseudopotentials of the elements. Interaction potentials of core electrons were replaced by the Projector-Augmented Wave (PAW) method as parameterized in the Vienna ab initio simulation package (VASP)^51–53^. The Hubbard *U* parameter (GGA+*U*) was adopted to enhance the description of correlation effects and to alleviate the self-interaction error^54^. The optimized effective interaction parameter *U*_eff_ (*U*_eff_ = *U*−*J*) of 3.32 eV was used for Co transition metal which has been widely adopted in the Materials Project^55^. All atoms and cell parameters of each model structure were fully relaxed until the total energy was approached within 10^−4^ eV. A gamma point mesh was performed with 2×2×1 k-points for YBaCo_4_O_7_ (110) slab models to sample the Brillouin zone. We used the tetrahedron smearing method with Blöchl corrections^51^. The periodic boundary condition was imposed on the unit cell where a vacuum size was almost twice larger (> 20 Å) than the thickness of YBaCo_4_O_7_ (110)’s slab to prohibit interactions between the top and bottom surface.

#### Surface structure calculation

The Gibbs energy of different surface termination was calculated with the equation of

$${{{{{\rm{\sigma }}}}}}=\frac{1}{A}\left[{E}_{{{{{{{\rm{slab}}}}}}}}-n{E}_{{{{{{{\rm{bulk}}}}}}}}\right]$$

where *E*_slab_ is the energy of slab, *E*_bulk_ is the energy of bulk YBC4 per formula unit, *n* is the number of the formula unit in the slab, *A* is the surface area. All the surface atoms were fully relaxed during the surface structure optimization.

#### Wulff structure simulation

The simulation of Wulff shapes was done with the software SOWOS, an open-source program for the three-dimensional Wulff construction^56^. The simulated Wulff structure of YBC4 that matched the corresponding relative surface energies as listed in Fig. S4. In comparison with that of the (001) and (111) facets, the surface energies of the (110) facet decreased rapidly.

#### Crystal orbital overlap calculation

The crystal overlap population scheme of the LOBSTER method (version 2.2.1) was employed to study bonding and chemical interaction properties^57^. The “completeness” of the basis functions in representing the valence electronic configuration of the POTCAR files used in the VASP calculations was verified with a small absolute charge spilling value of 0.56%–0.83%. To match the number of bands in the VASP calculations with the number of orbitals used in the LOBSTER projection scheme, the VASP input parameter NBANDS was set to 216 for all model systems. Symmetry was switched off (ISYM = −1) for all VASP calculations. Crystal orbital overlap population (COOP) is very powerful in understanding chemical bonding by showing the bonding (COOP > 0) and antibonding (COOP < 0) character of each studied chemical bond.

#### Calculation of OER free energy

The reaction pathway of OER on the surface of YBC was analyzed by considering all the possible reaction intermediates. In the YBC4 (110) facet, the cobalt-terminated surface enables the exposure of adjacent cobalt sites. The case that all the surface cobalt sites are occupied by an adsorbed *OH species was chosen as the starting point of OER. For each elementary step, the reactant OH^−^ could attack either the Triangular or the Kagome cobalt site. Therefore, both cases need to be evaluated and the thermodynamically more feasible one was considered as the real elementary step. The free energy change for each step was defined as the change of chemical potentials between the products and the reactants. Taking the elementary step of

$$\begin{array}{c}\left(\begin{array}{c}\triangle \\ k\end{array}*{{{{{\rm{OH}}}}}}\, \cdots \begin{array}{c}\triangle \\ t\end{array}*{{{{{\rm{OH}}}}}}\right)+{{{{{{\rm{OH}}}}}}}^{-}\to \left(\begin{array}{c}\triangle \\ k\end{array}*{{{{{\rm{O}}}}}}\,\cdots \begin{array}{c}\triangle \\ t\end{array}*{{{{{\rm{OH}}}}}}\right)+{\rm {H}}_{2}{\rm {O}}+{{{{{{\rm{e}}}}}}}^{-}\end{array}$$

as an example, the free energy change is calculated as

$$\triangle G={\mu }\left(\begin{array}{c}\triangle \\ k\end{array}*{{{{{\rm{O}}}}}}\,\cdots \begin{array}{c}\triangle \\ t\end{array}*{{{{{\rm{OH}}}}}}\right)+{\mu}({{{{{\rm{H}}}}}}_{{{{{\rm{2}}}}}}{{{{{\rm{O}}}}}})-{\mu} \left(\begin{array}{c}\triangle \\ k\end{array}*{{{{{\rm{OH}}}}}}\,\cdots \begin{array}{c}\triangle \\ t\end{array}*{{{{{\rm{OH}}}}}}\right)-{\mu}({{{{{{\rm{OH}}}}}}}^{-})$$

The chemical potentials of each species were calculated by standard DFT techniques, as described by Peterson et al.^40^. Solvation correction was included in the initial state where two terminal OH were adsorbed on Co tetrahedra to address the stabilization of hydroxyl adsorbates.

## Supplementary information

Supplementary Information

## Data Availability

The data that support the findings of this study are provided in the Main Text and Supplementary Information.

## Code availability

The analysis codes that support the findings of the study are available from the corresponding authors upon reasonable request.
