# Supplementary material for: Enhanced oxygen evolution over dual corner-shared cobalt tetrahedra
Source: Nat Commun. 2022 Sep 20;13:5510. doi: 10.1038/s41467-022-33000-w (PMC9489709; doi:10.1038/s41467-022-33000-w)
Supplement: Supplementary file 1 — Supplementary Information [file 41467_2022_33000_MOESM1_ESM.pdf]

Supplementary Information

Enhanced oxygen evolution over dual corner-shared cobalt tetrahedra

Yubo Chen [a,b,c,d Δ], Joon Kyo Seo [e,f Δ], Yuanmiao Sun [a Δ], Thomas A. Wynn [e,f], Marco Olguin [e,f], Minghao Zhang [e,f], Jingxian Wang [a], Shibo Xi [g], Yonghua Du [g], Kaidi Yuan [h], Wei Chen [h], Adrian C. Fisher [b,i], Maoyu Wang [j], Zhenxing Feng [j], Jose Gracia [k], Li Huang [l], Shixuan Du [l], Hong-Jun Gao [l], Ying Shirley Meng [e,f,m], Zhichuan J. Xu [a,b,c,d]**

[a] *School of Material Science and Engineering, Nanyang Technological University, 50 Nanyang Avenue, Singapore 639798, Singapore*

[b] *The Cambridge Centre for Advanced Research and Education in Singapore, 1 CREATE way, Singapore 138602, Singapore*

[c] *Solar Fuels Laboratory, Nanyang Technological University, 50 Nanyang Avenue, Singapore 639798, Singapore*

[d] *Energy Research Institute @ Nanyang Technological University, 50 Nanyang Avenue, Singapore 639798, Singapore*

[e] *Department of Nano Engineering, University of California San Diego, 9500 Gilman Drive, La Jolla, CA 92093, USA*

[f] *Materials Science and Engineering, University of California San Diego, 9500 Gilman Drive, La Jolla, CA 92093, USA*

[g] *Institute of Chemical and Engineering Sciences, A*STAR,1 Pesek Road, 627833, Singapore*

[h] *Department of Physics, National University of Singapore, 2 Science Drive 3, 117542 Singapore*

[i] *Department of Chemical Engineering, University of Cambridge, Cambridge, CB2 3RA, UK.*

[j] *School of Chemical, Biological, and Environmental Engineering, Oregon State University, Corvallis, OR 97331, USA*

[k] *MagnetoCat SL, General Polavieja 9 3I, Alicante, 03012 Spain*

[l] *Beijing National Laboratory for Condensed Matter Physics and Institute of Physics, Chinese Academy of Science, Beijing 100190, China*

[m] *Pritzker School of Molecular Engineering, University of Chicago, Chicago, IL 60637, USA*

[Δ] These authors contributed equally.

E-mail: xuzc@ntu.edu.sg; shmeng@ucsd.edu, shirleymeng@uchicago.edu

Contents

Supplementary Figures ... 5

Fig. S1 Synchrotron-based diffraction data of YBC4 ... 6

Fig. S2 HR-TEM images of YBC4 ... 7

Fig. S3 XANES profiles of YBC4, Co_3O_4, and CoO ... 8

Fig. S4 Wulff structure of YBC4 ... 9

Fig. S5 Calculated COOP for Co-O bonds ... 10

Fig. S6 HT-XPS spectra of Y 3d and Ba 4d ... 11

Fig. S7 HT-XPS spectra of C 1s ... 12

Fig. S8 High angle annular dark field STEM ... 13

Fig. S9 EELS spectra of Co L-edge and Ba M-edge ... 14

Fig. S10 Distances between the Co-L_3 and the Co-L_2 white lines ... 15

Fig. S11 EELS spectra of O K-edges ... 16

Fig. S12 DOS of bulk YBC4 ... 17

Fig. S13 PDOS of Co from the active site at the YBC4 (110) surface ... 18

Fig. S14 Rietveld refined XRD patterns ... 19

Fig. S15 Nitrogen adsorption-desorption isotherm curves ... 20

Fig. S16 Initial 5 cycles of CVs ... 21

Fig. S17 Faradic efficiency ... 22

Fig. S18 Tafel plots ... 23

Fig. S19 1000 CV cycles of YBC4 and BSCF ... 24

Fig. S20 TEM images of surface structure ... 26

Fig. S21 Fitted Co 2p core spectra ... 27

Fig. S22 XRD patterns of YBC4 before and after thermal/electrochemical oxidation ... 28

Fig. S23 Oxidation of perovskite ... 30

Fig. S24 XRD patterns of YBC4 before and after electrochemical oxidation ... 31

Fig. S25 Co K-edge EXAFS spectra of different YBC4 samples ... 32

Fig. S26 OER currents for as-prepared YBC4 and Thermally reduced YBC4 ... 33

Fig. S27 Calculated possible paths in the OER on the YBC4 (110) surface ... 34

Fig. S28 Local structures in YBC4 ... 35

Fig. S29 Interatomic distances ... 36

Fig. S30 Another two mono-μ-oxo-bridged Co ... 37

Fig. S31 The formation of "tri-oxygen-coordinated cobalt" 38
Supplementary Tables 39
Table S1 Refined structure information. 40
Table S2 Detailed parameters from XPS fitting of O 1s, Y 3d, and Ba 4d. 41
Table S3 ICP-MS results from used electrolytes after potential cycling. 42
Table S4 Detailed parameters from XPS fitting of Co 2p. 43
Table S5 Fitting parameters of the Fourier-transformed k^3-weighted Co K-edge EXAFS from YBC4 samples. 44
Table S6 Estimated values of key parameters from YBC4, $SrCoO_3$, and CoOOH. 45
Supplementary Discussion 46
A detailed comparison between Co tetrahedra and octahedra 47
The number of d electrons 47
Charge-transfer energy (CTE) 48
The number of e_g electrons 48
Geometry structure 49
Additional discussion of another two mono-μ-oxo-bridged Co 54
The effect of electrochemical oxidation 55

Supplementary Figures

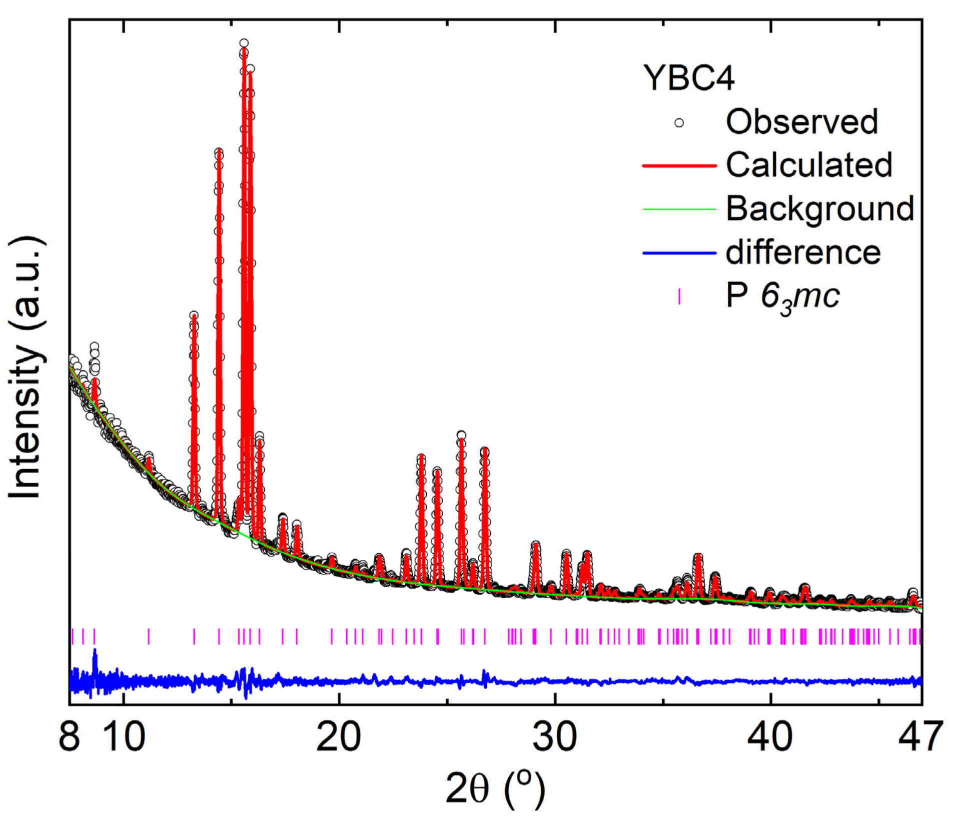

Fig. S1 Synchrotron-based diffraction data of YBC4 | Rietveld refinement of synchrotron-based diffraction data of as-prepared YBC4 at room temperature. Detailed structure parameters are presented in **Table S1**.

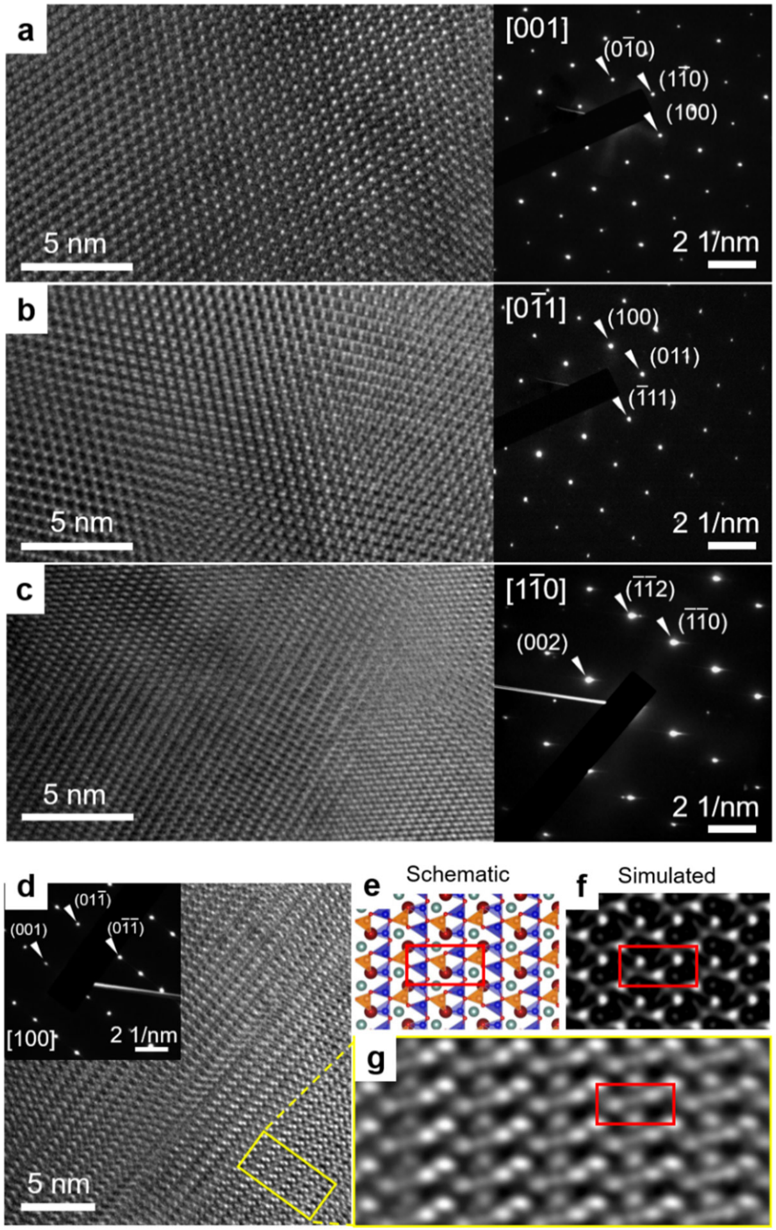

Fig. S2 HR-TEM images of YBC4 | The HR-TEM images of YBC4 along four different zone axes of [001] (**a**), [0$\bar{1}$1] (**b**), [1$\bar{1}$0] (**c**), and [100] (**d**). The corresponding SAED images are also presented and are indexed based on the refined lattice parameters from XRD refinement. (**e**) Schematic viewing from [100] direction. The triangular layers and Kagome layers are alternatively arranged. (**f**) Simulated image of (**e**) from JEMS software. (**g**) HRTEM images after FFT and inverse FFT processing alone [100] zone axes. The nearly identical atomic arrangement between the simulated image (**f**) and the enlarged HRTEM image (**g**) confirms the tetrahedral coordination of cobalt.

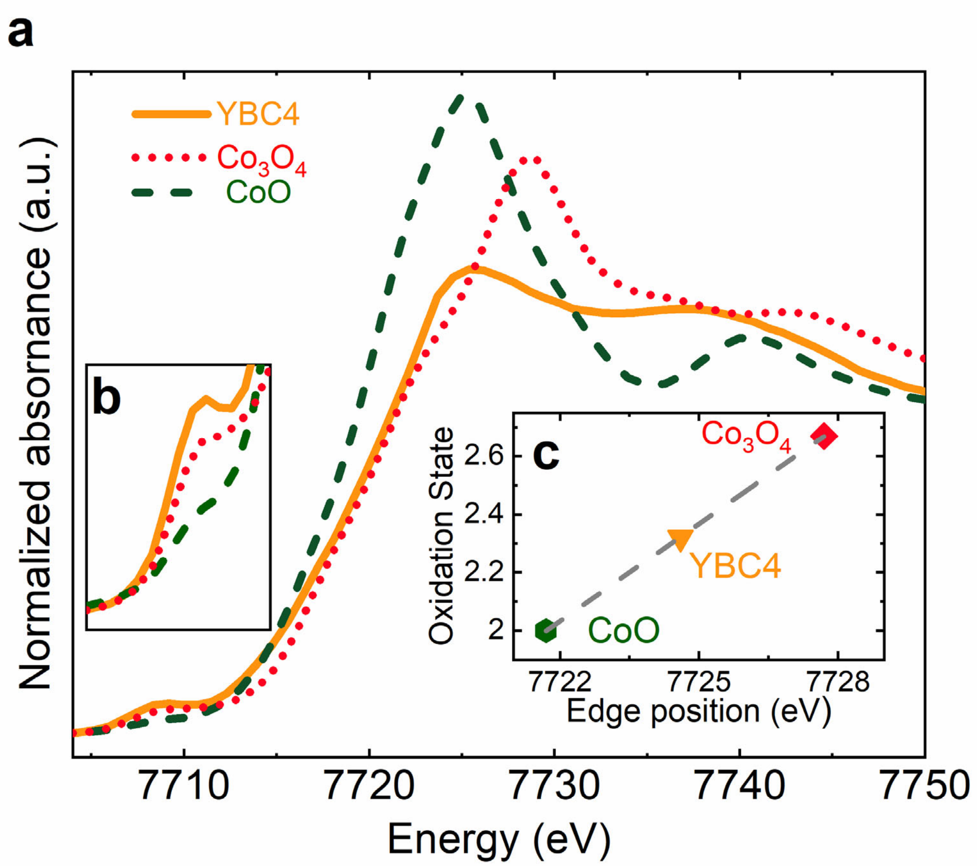

Fig. S3 XANES profiles of YBC4, Co_3O_4, and CoO | (**a**) XANES profiles of YBC4, Co_3O_4, and CoO. (**b**) Enlarge pre-edge peaks from Co in YBC4, Co_3O_4, and CoO. Before the white lines, characteristic pre-edge peaks, which originate from a 1s-3d transition, can be observed for Co and their intensity will increase if Co is in the tetrahedron with non-centrosymmetric geometry.[1] An intense shape pre-edge peak at approximately 7708 eV can be observed in the Co K-edge from YBC4, which hints the Co-ions are dominant in tetrahedral coordination in as-prepared YBC4. (**c**) The average oxidation state for the Co in YBC4 is estimated to be ~2.3+ with the edge positions of CoO (Co^{2+}) and Co_3O_4 ($Co^{2.67+}$) as standards.

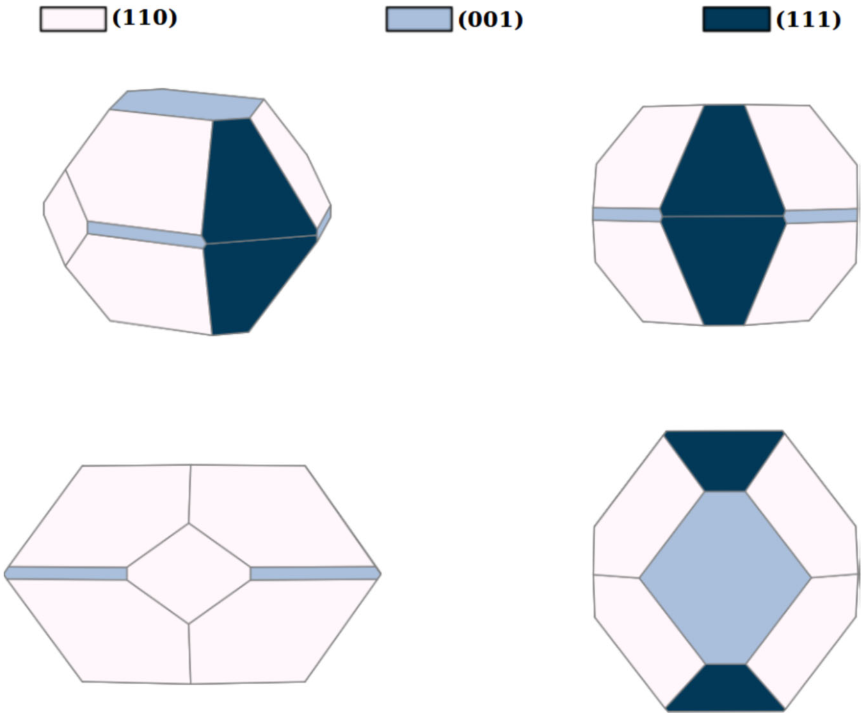

Fig. S4 Wulff structure of YBC4 | The generated Wulff structure of YBC4. Three facets of (110), (001), and (111) are optimized and the surface atoms are set to be fully relaxed during the optimization process. The surface energies of 62.520, 64.211, and 73.579 meV/Å^2 are obtained for (110), (001), and (111) facets, respectively. In the generated Wulff structure, the facet of (110) is found to be dominant.

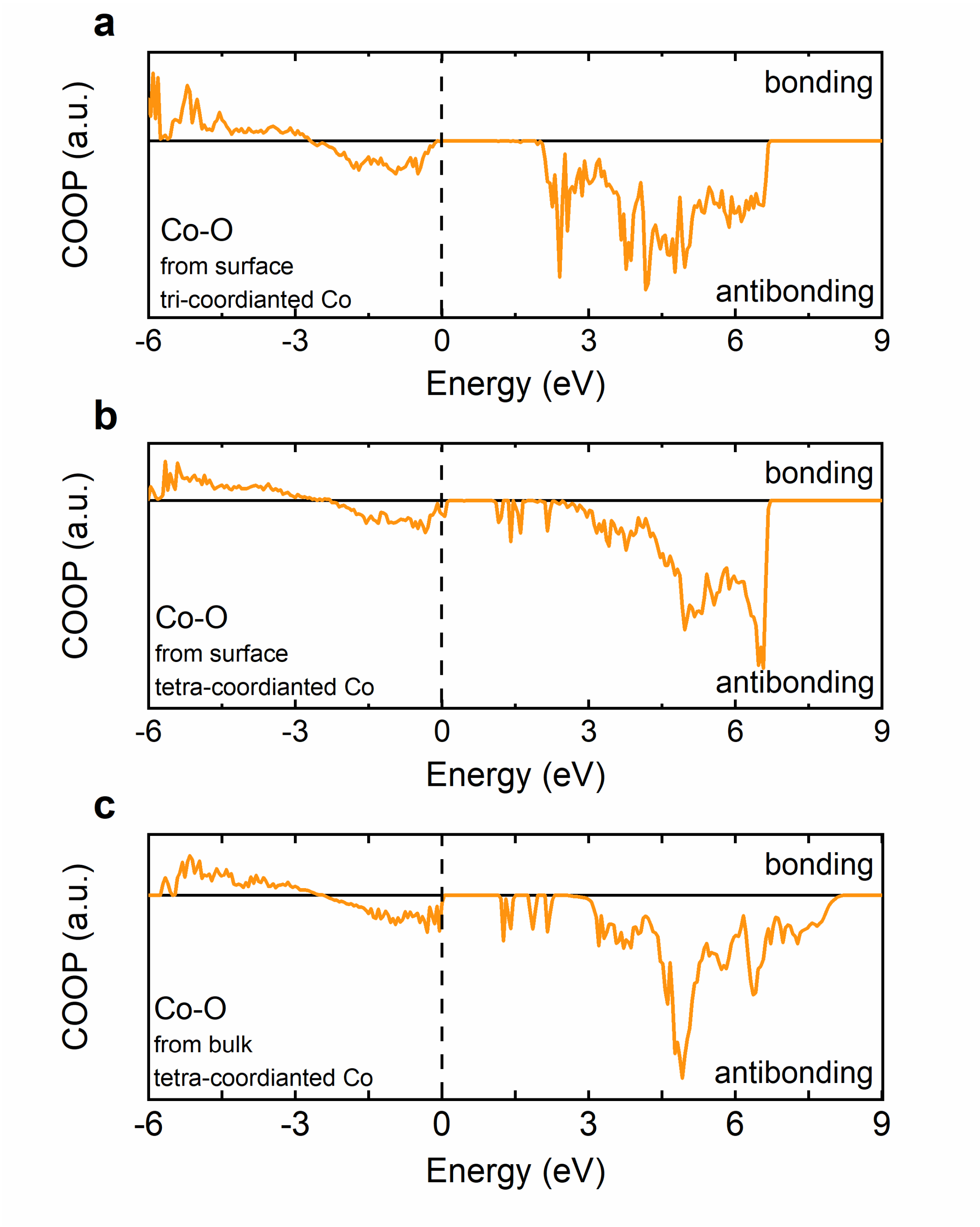

Fig. S5 Calculated COOP for Co-O bonds | Calculated COOP for Co-O bonds from the surface tri-coordinated Co (**a**), surface tetra-coordinated Co (**b**), and bulk tetra-coordinated Co (**c**) in YBC4. The Fermi level (E_f) is indicated by vertical dashed lines.

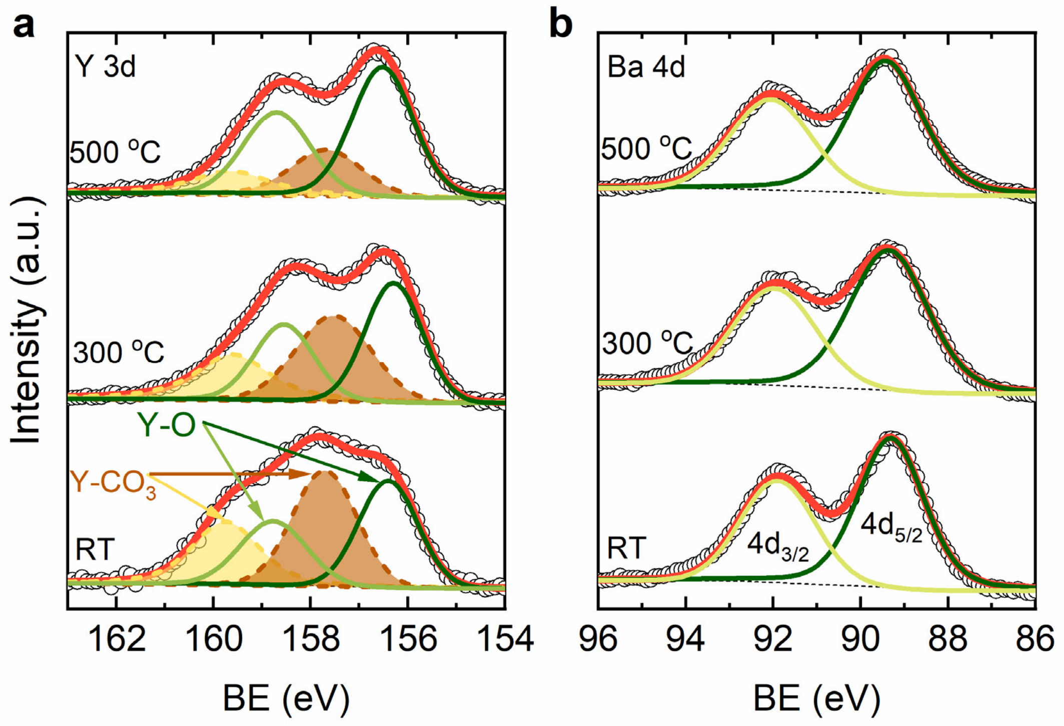

Fig. S6 HT-XPS spectra of Y 3d and Ba 4d | HT-XPS spectra of Y 3d (**a**) and Ba 4d (**b**) were obtained at RT, 300 ºC, and 500 ºC. The black circles were raw data, the red lines indicate the fit sum, the black dash lines are baselines, and the shadowed area in Y 3d spectra represents the signal of Y adsorbed with carbonate.

Two doublets from the Y 3d peaks can be obtained when performing the fit. The shadowed doublet at approximately 157.6 eV and 159.6 eV can be largely attributed to the Y bonded with carbonate.[2,3] The intensity of the first doublet gradually decreased, indicating the desorption of carbonate from Y. The second doublet (~156.4 and ~158.7) was related to the Y bonded with O.[2,4] As was expected, no changes for this second doublet can be observed as the temperature increased. From the Ba 4d XPS spectra, only one doublet at approximately 89.4 eV ($4d_{5/2}$) and 92 eV ($4d_{3/2}$) can be observed and can be perfectly fitted with a relative area ratio of 3:2 for the doublet. No additional doublets can be observed in all cases. Thus, the doublet should mainly contribute from Ba-O.[5] Based on these results, the detected OH groups (**Fig. 1c**) should bond with the surface Co.

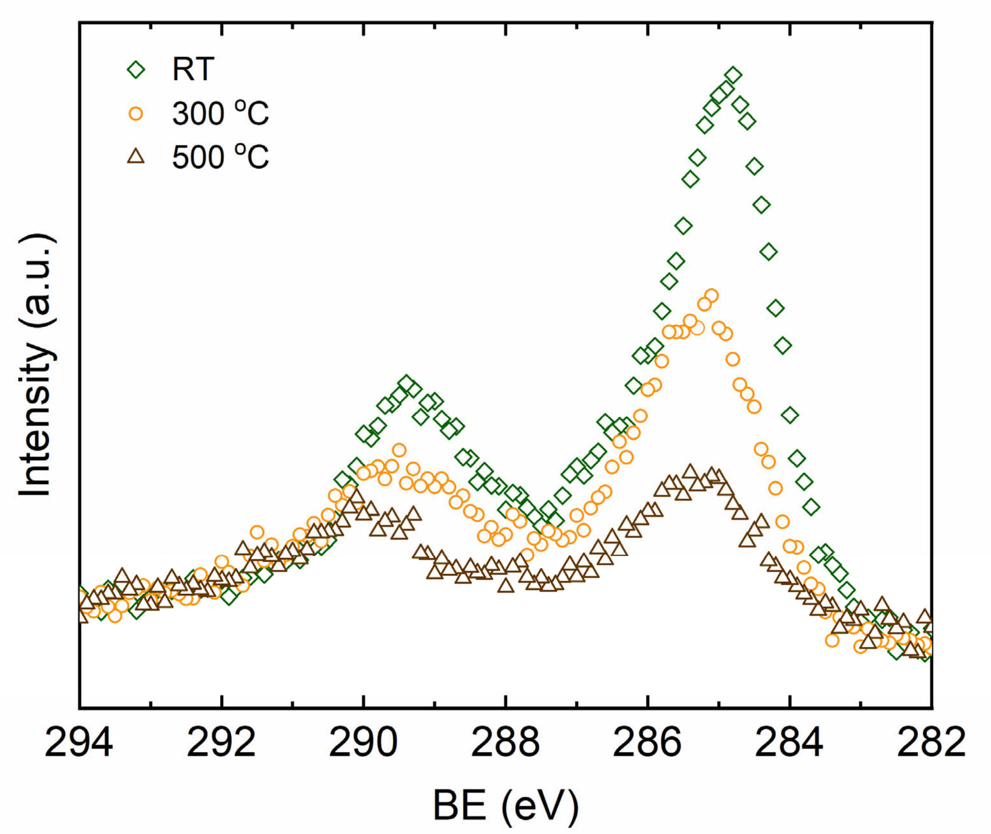

Fig. S7 HT-XPS spectra of C 1s | HT-XPS spectra of C 1s obtained at RT, 300 ºC, and 500 ºC. From C1s spectra, the intensity of the peaks (in between 289 eV and 290 eV) from carbonate and adventitious carbon contamination (~285 eV) steeply decrease after high-temperature thermal treatment.

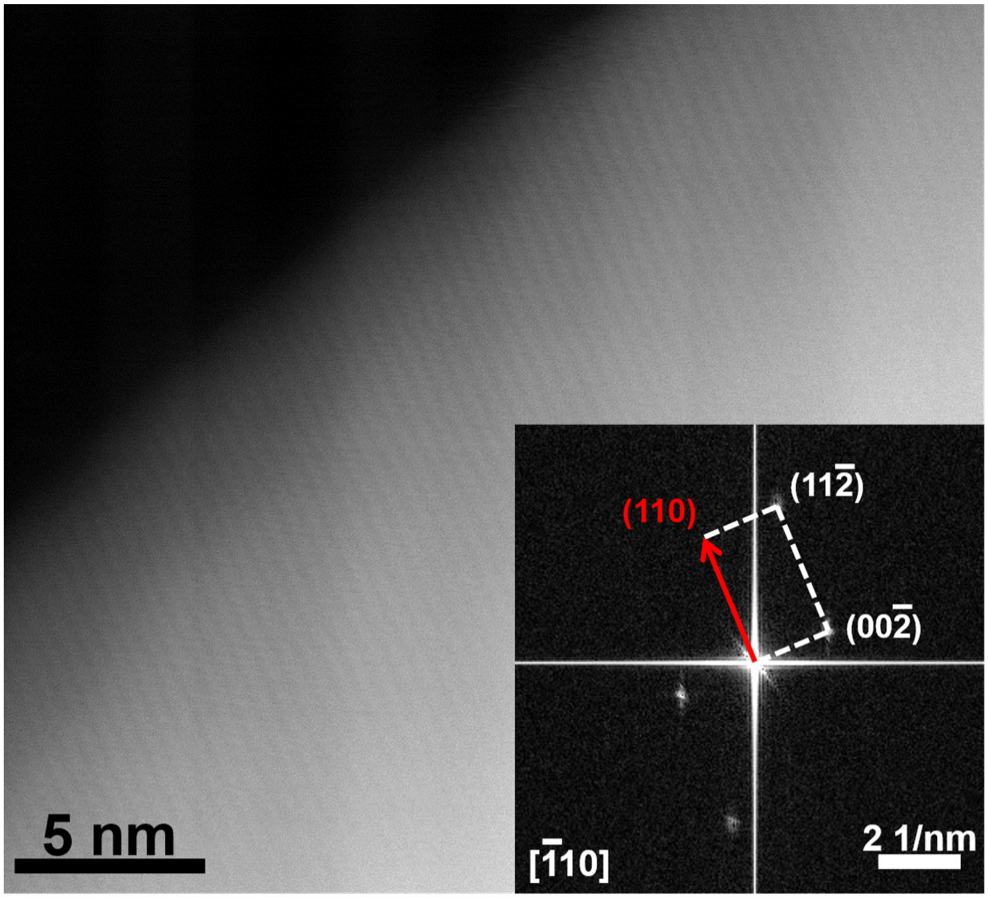

Fig. S8 High angle annular dark field STEM | A high angle annular dark field (HAADF) STEM image of (110)-plane exposed surface. The corresponding FFT image is presented as the inset and the (110) orientation is marked with a red arrow.

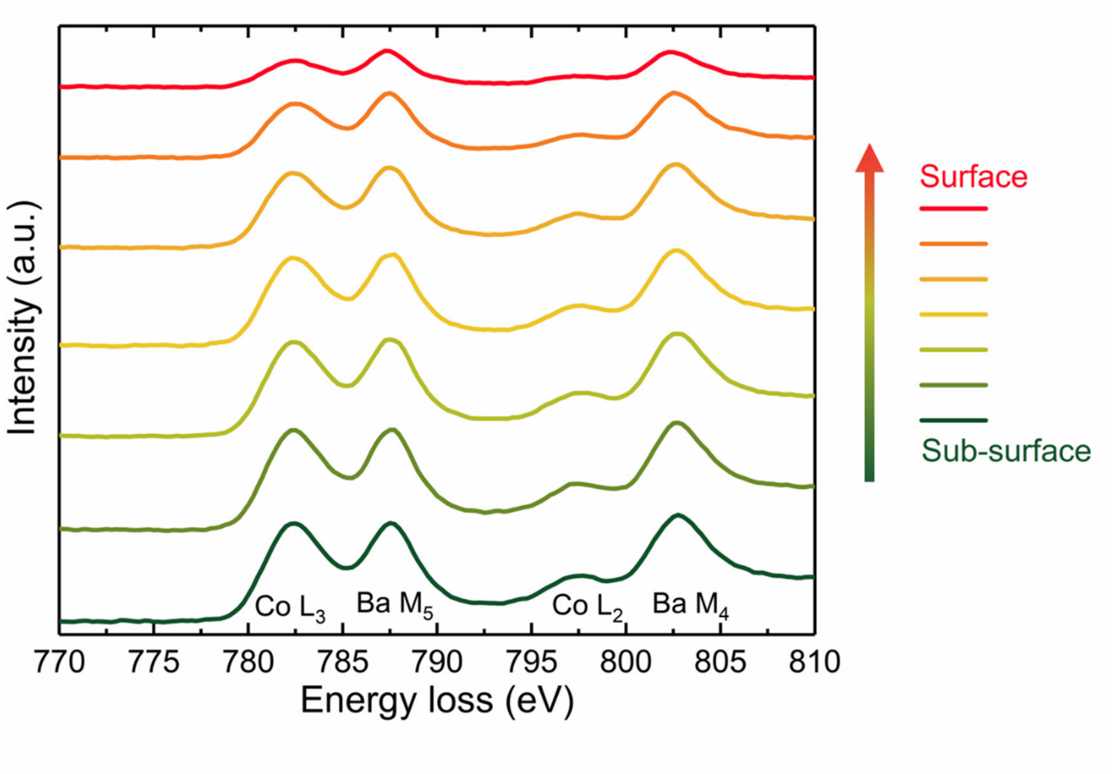

Fig. S9 EELS spectra of Co L-edge and Ba M-edge | The EELS spectra of Co L-edge and Ba M-edge from sub-surface to surface.

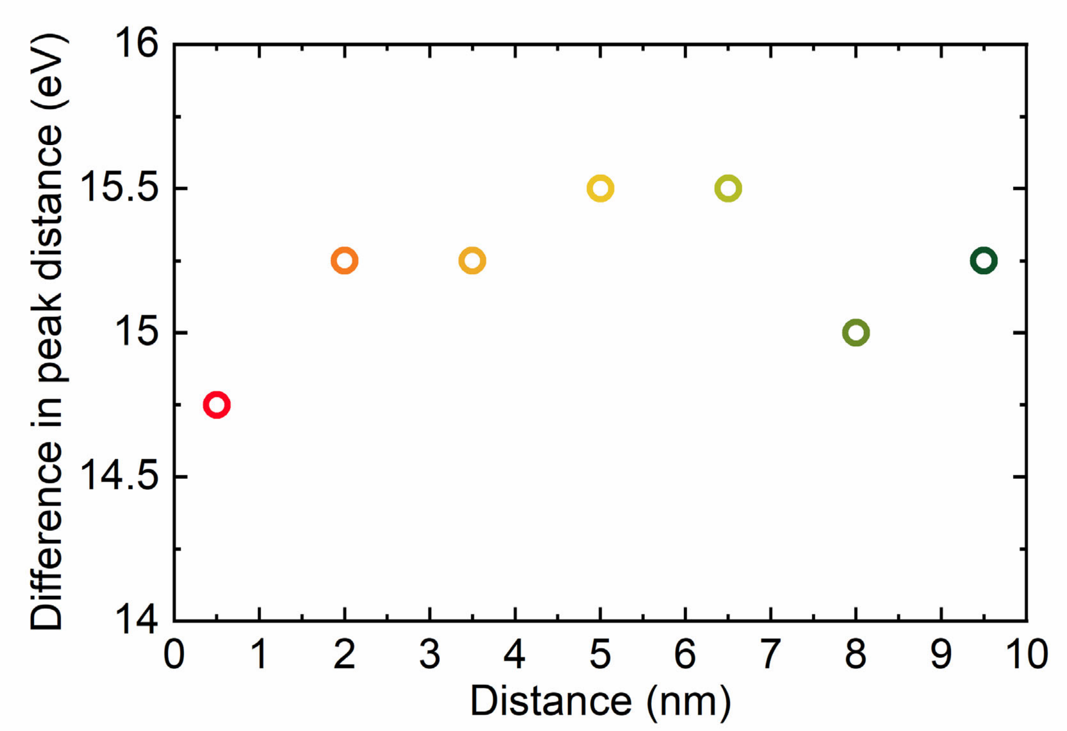

Fig. S10 Distances between the Co-L_3 and the Co-L_2 white lines | Measured distances between the Co-L_3 and the Co-L_2 white lines. According to the previous study, distances of 14.75 eV and 15.5 eV correspond to an average Co valence state of ~2.3+ and ~2.0+, respectively.[6]

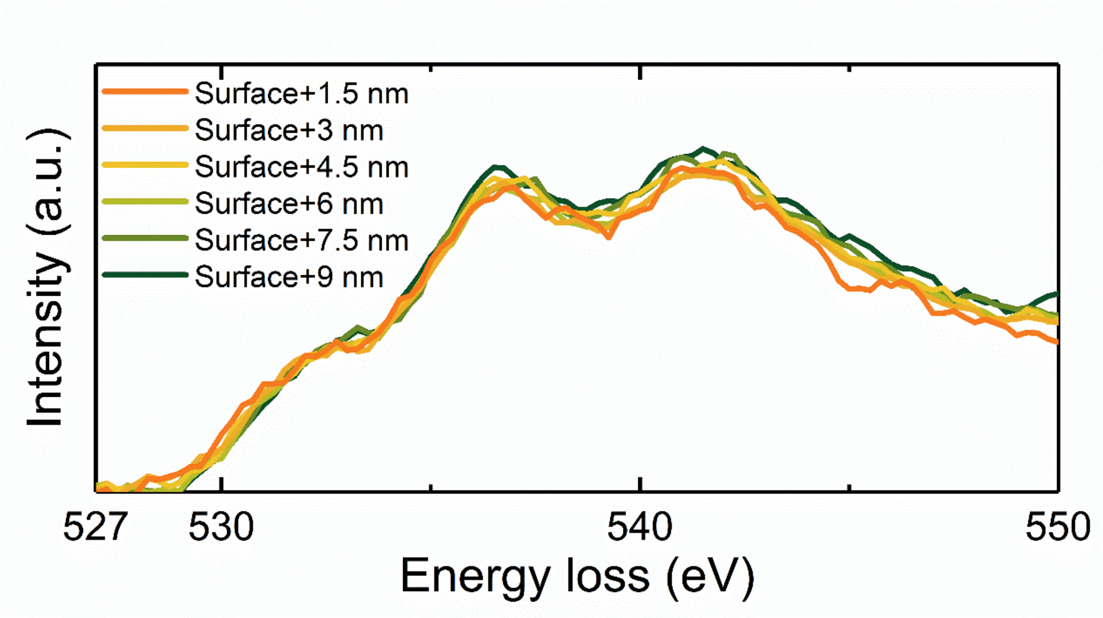

Fig. S11 EELS spectra of O K-edges | EELS spectra of O K-edges from the sub-surface regions with different depths.

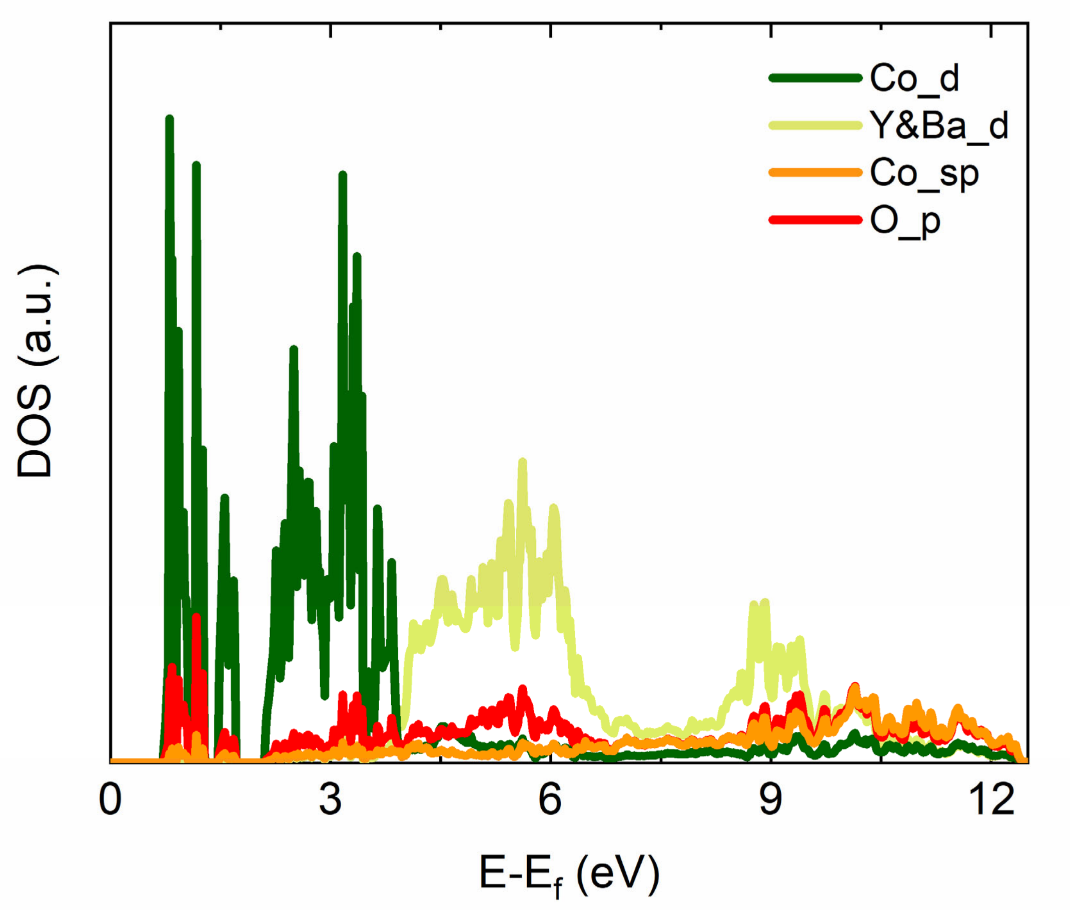

Fig. S12 DOS of bulk YBC4 | The calculated DOS of bulk YBC4. Above the Fermi level, the O 2p orbital hybridized with the Co 3d, Y 4d&Ba 5d, and Co 4sp, corresponding to the observed α, β, and γ peaks in the EELS spectra (**Fig. 1d** of the main text).

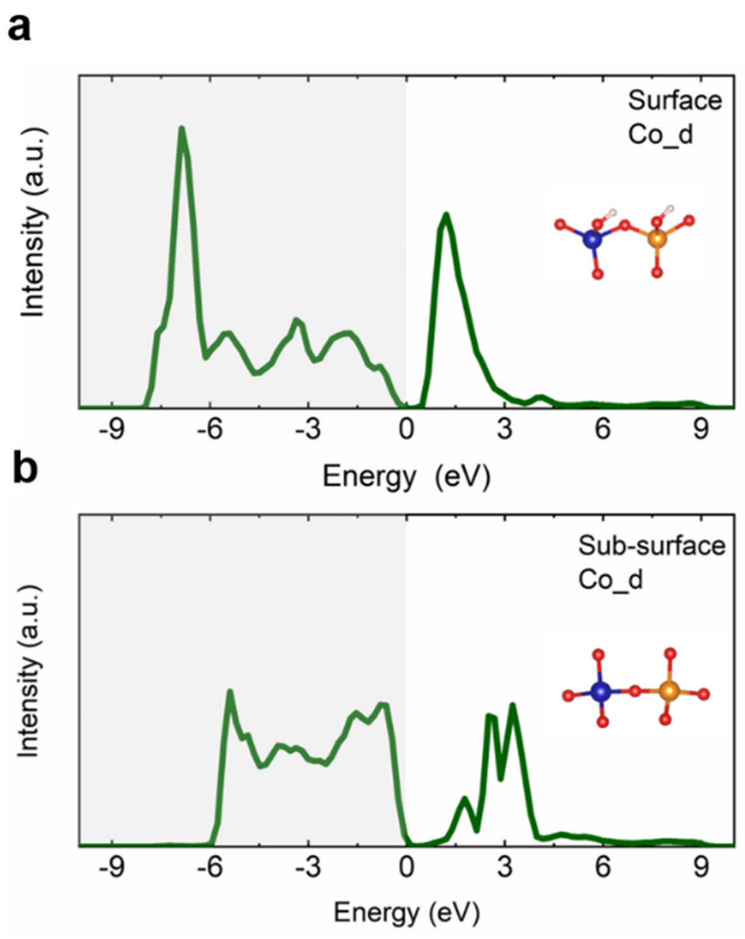

Fig. S13 PDOS of Co from the active site at the YBC4 (110) surface | The PDOS of Co from the active site at the YBC4 (110) surface (**a**) and the YBC4 sub-surface (**b**). the corresponding local structures of Co are shown in the insets. Two representative cobalt in tetrahedral geometry are included, the blue one is from the Kagome layer and the orange one is from the Triangular layer. The shadowed regions (below Fermi level) are used for estimating the number of d electrons. From **Fig. S13a**, the number of d orbital electrons is estimated to be 6.9, indicating an oxidation state of ~2.1+ for Co over the surface. From **Fig. S13b**, the number of d orbital electrons is estimated to be 7.1, indicating an oxidation state of ~1.9+ for Co in the sub-surface. The valence state of cobalt cations estimated using PDOS is consistent with the dominant divalent state of cobalt cations estimated using EELS spectra (**Fig. S10**).

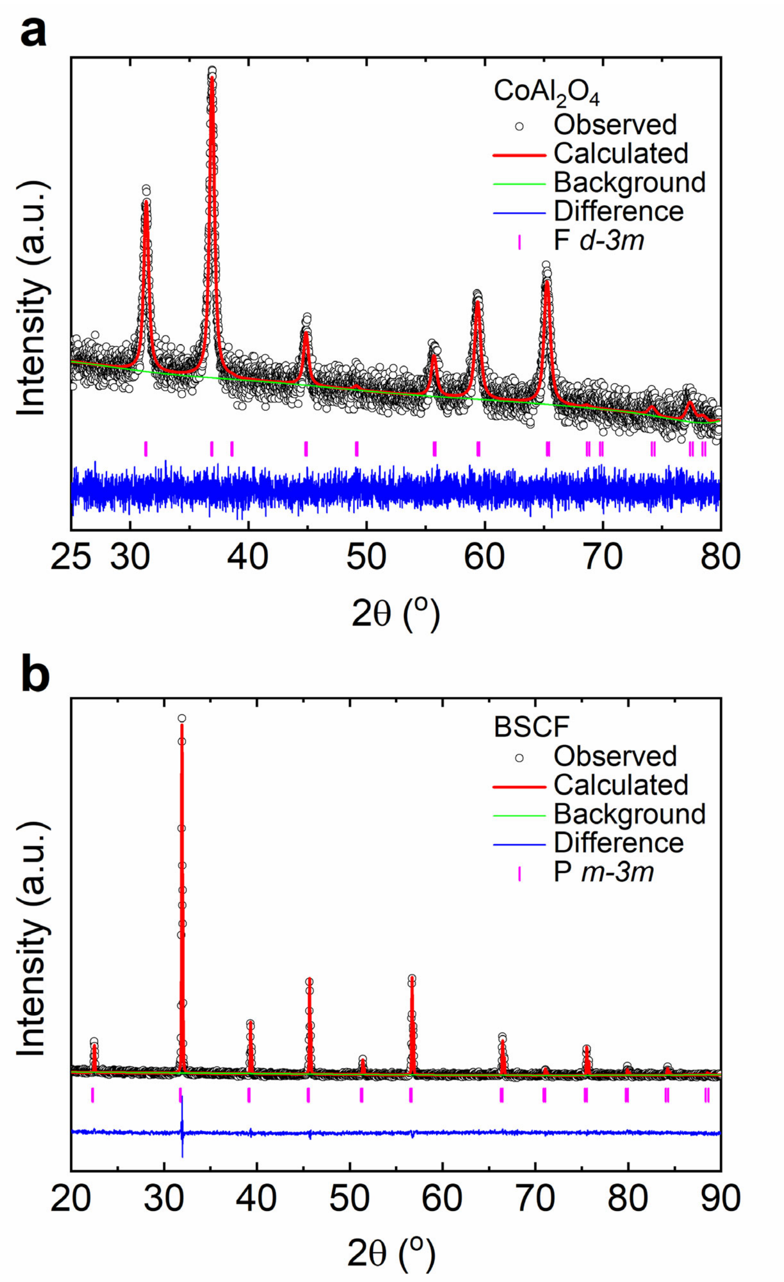

Fig. S14 Rietveld refined XRD patterns | Observed XRD patterns and Rietveld refined results for as-prepared (**a**) $CoAl_2O_4$ and (**b**) BSCF. The reliability factors for the $CoAl_2O_4$ are R_{wp}=1.69%, R_p=1.34%, and x^2=1.039; The reliability factors for the BSCF are R_{wp}=5.02%, R_p=4.01%, and x^2=1.491. During the refinement of the pattern from $CoAl_2O_4$, the attempt to refine Al in the tetrahedral site (8d) is unsuccessful, confirming that the Co-ions in as-synthesized $CoAl_2O_4$ are dominant in tetrahedral sites.

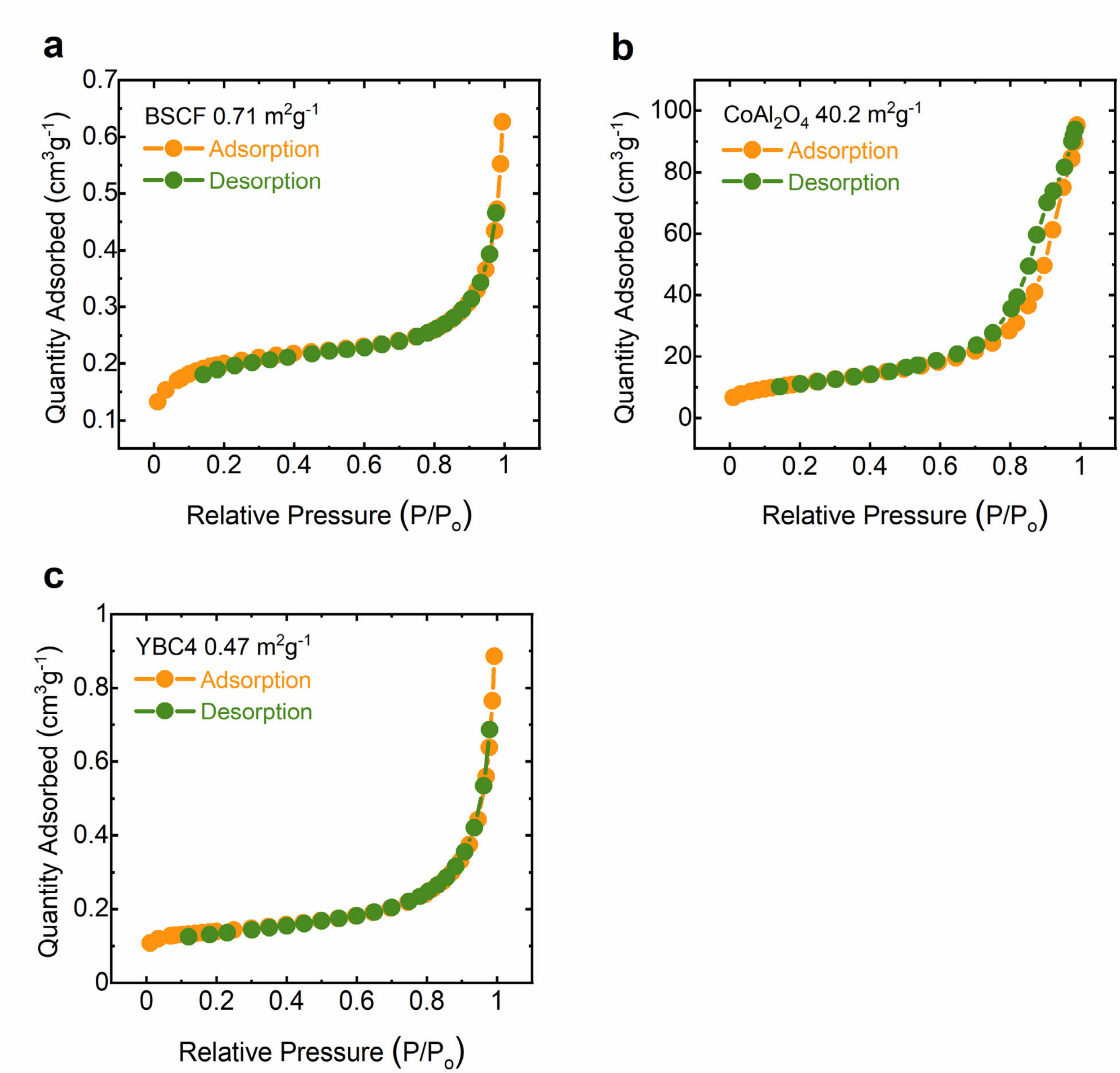

Fig. S15 Nitrogen adsorption-desorption isotherm curves | The Nitrogen adsorption-desorption isotherm curves of (**a**) BSCF, (**b**) $CoAl_2O_4$, and (**c**) YBC4 catalysts. A surface area of 4.9 m^2g^{-1} was applied for IrO_2 from Sigma-Aldrich.[7]

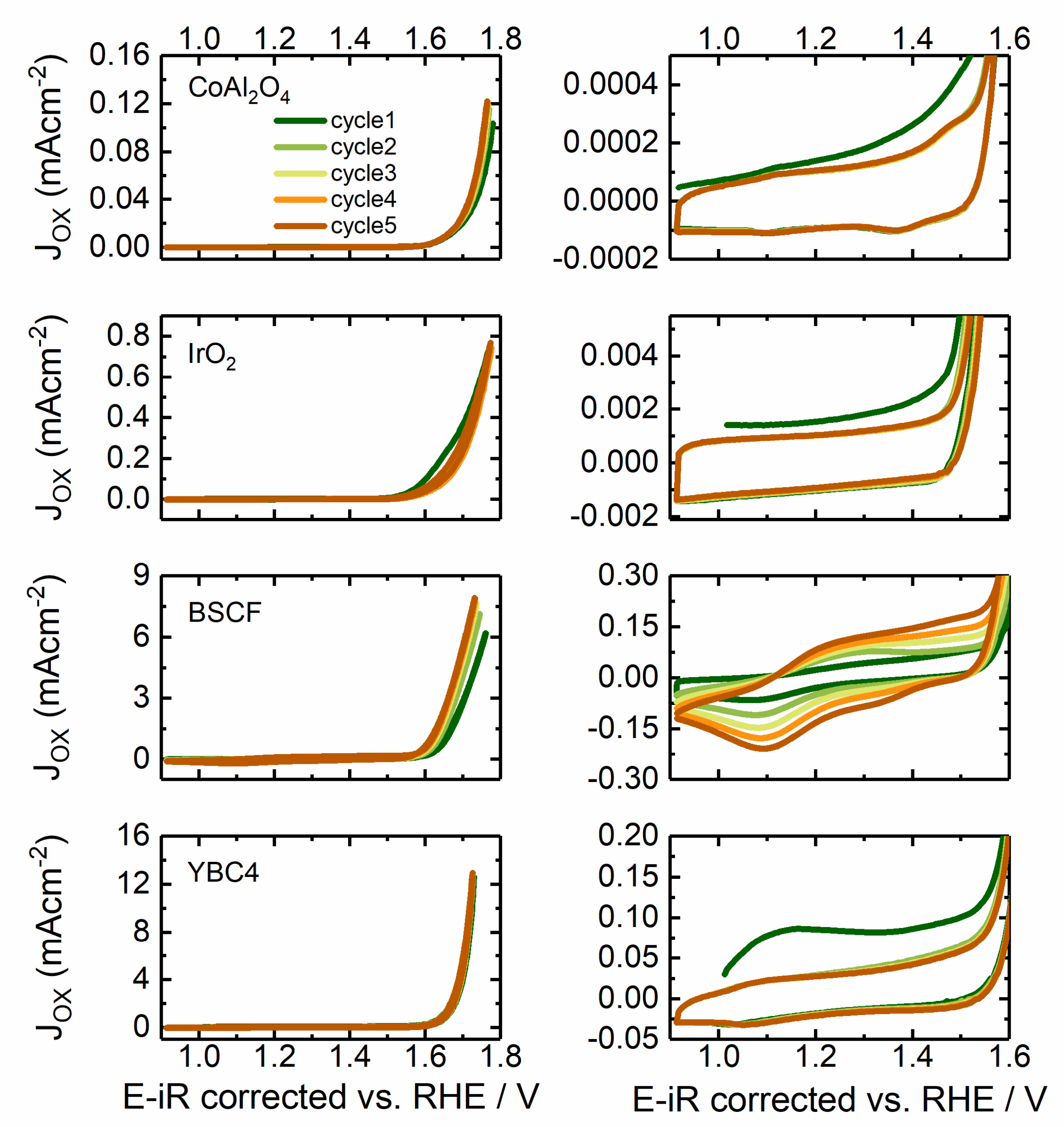

Fig. S16 Initial 5 cycles of CVs | Initial 5 cycles of CVs for $CoAl_2O_4$, IrO_2, BSCF, and YBC4. Note that the BSCF shows a distinctive performance improvement as well as increased capacitance, which is related to its unstable surface structure.[8,9]

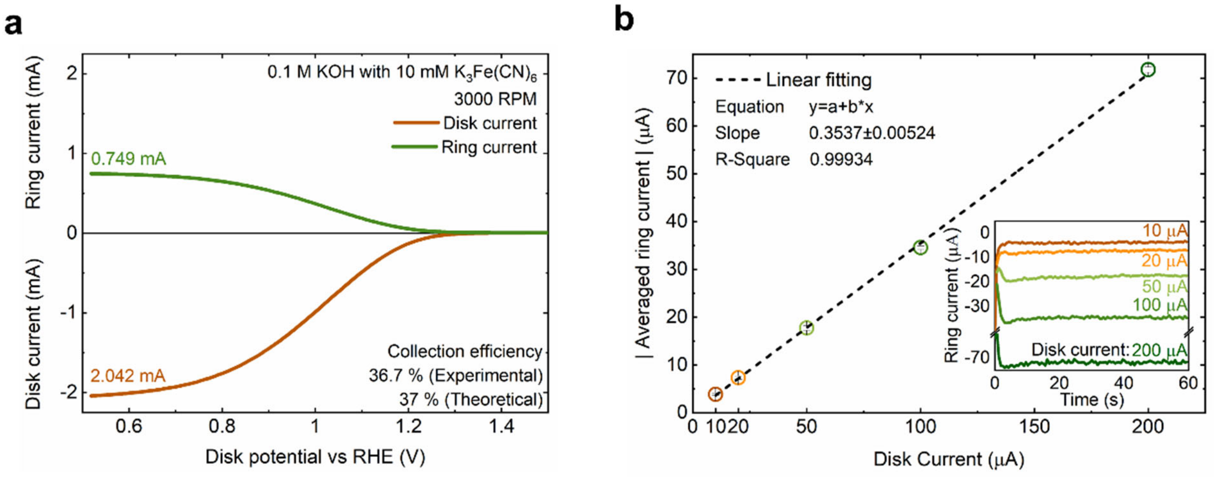

Fig. S17 Faradic efficiency | (**a**) Rotating ring-disk electrode voltammogram at a rotation rate of 3000 rpm. (**b**) Linear fitting of the measured disk currents and the average ring currents (absolute values). The inset shows the measured ring currents at different disk currents.

As shown in **Fig. S17a**, the experimental collection efficiency of the used RRDE is measured using a well-defined redox system, i.e., the redox of ferricyanide/ferrocyanide in potassium ferricyanide ($K_3Fe(CN)_6$). The experimental collection efficiency is calculated based on the measured disk and ring current during the redox of ferricyanide/ferrocyanide. An experimental collection efficiency of 36.7 % is obtained, which is close to the theoretical efficiency of 37 %.

The evolved oxygen from the disk electrode (loaded with YBC4 catalyst) is detected with the Pt-ring electrode, on which a four-electron oxygen reduction reaction occurs.[10]

As shown in the inset of **Fig. S17b**, chronopotentiometry is applied, and the disk currents change sequentially from 10 μA to 200 μA. The corresponding ring currents are collected with a duration of 60 s. The average ring current is calculated by averaging the measured ring currents from 10 s to 60 s. With a linear fitting of the measured disk currents and the average ring currents (absolute values), the faradic efficiency can be estimated with the following equation:

$$F = \frac{\boldsymbol{I_{OER}}}{\boldsymbol{I_{disk}}} = \frac{(I_{ring} - i_a)/N_{exp}}{I_{disk}} = \frac{(I_{ring} - i_a)/I_{disk}}{N_{exp}} = \frac{\boldsymbol{b_{slope}}}{\boldsymbol{N_{exp}}}$$

Where I_{OER} is the current from oxygen evolution, I_{disk} is the measured disk current, I_{ring} is the measured ring current (absolute value), i_a is the background current in the ring disk, N_{exp} is the experimental collection efficiency, and b_{slope} is the slope from linear fitting (**Fig. S17b**).

A Faradic efficiency of 96.5±1.4 % is obtained from the disk electrode with YBC4 catalyst.

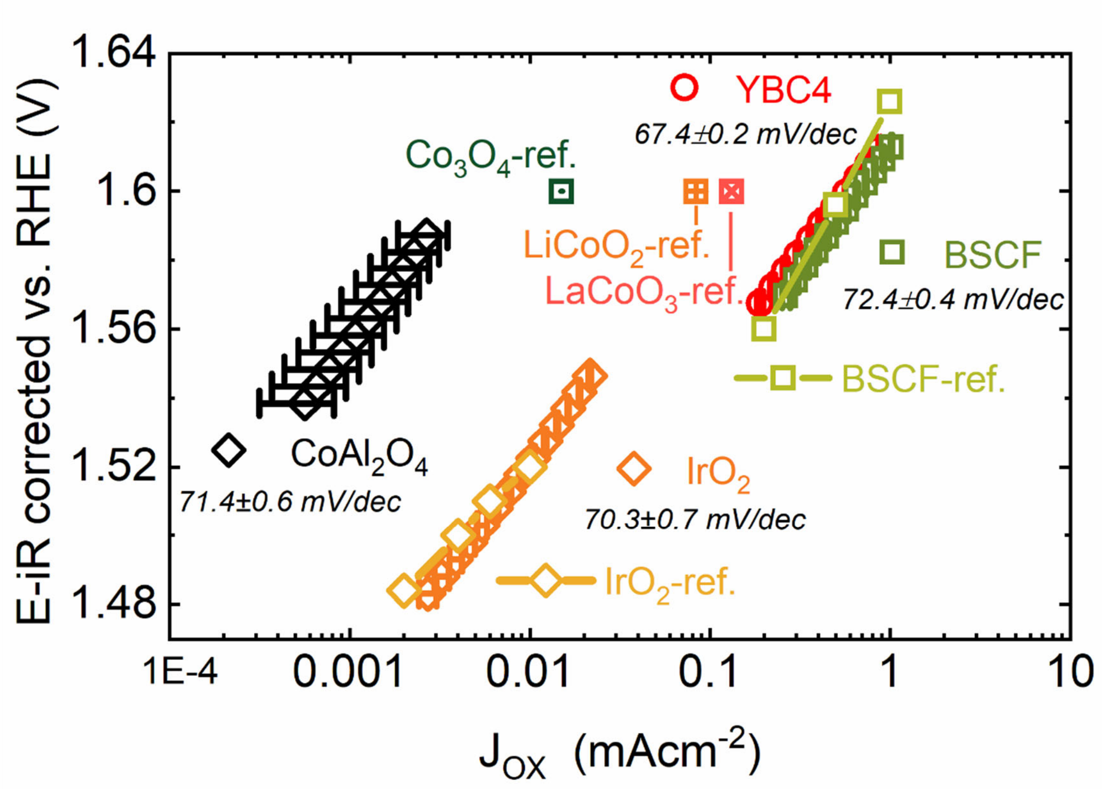

Fig. S18 Tafel plots | Tafel plots of different samples. Reported activities of IrO_2, BSCF, Co_3O_4, $LiCoO_2$, and $LaCoO_3$ are also presented.[10-14] Caution that different activities have been reported with BSCF materials.[8,12,15] For instance, the activity reported by Suntivich et al. is superior to our results.[12] This difference has been attributed to different synthetic methods.[15] The performance of BSCF reported in our study is much close to the reported activity of BSCF synthesized with a similar method.[11,15,16] Thus, it is reasonable to compare the activity between YBC4 and BSCF in our study as the identical synthetic method is applied for these two materials.

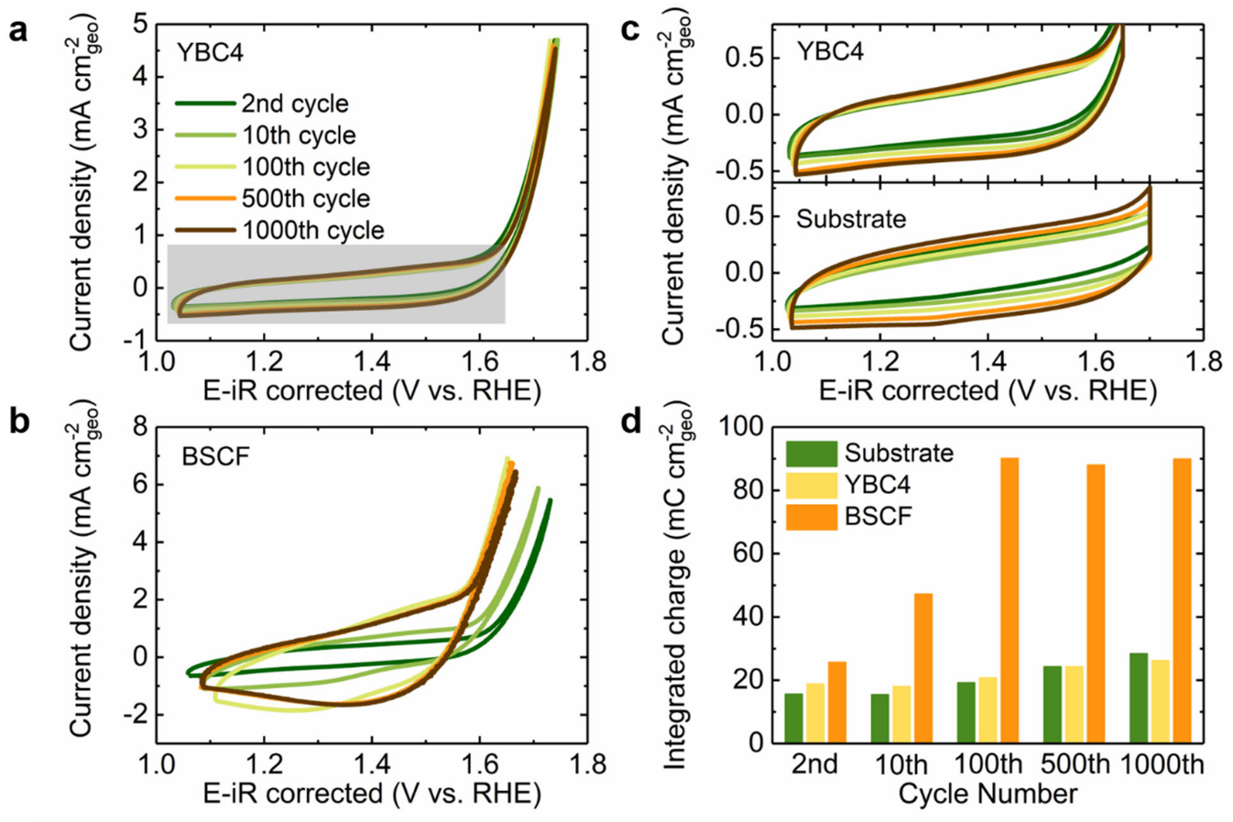

Fig. S19 1000 CV cycles of YBC4 and BSCF | CVs of YBC4 (**a**) and BSCF (**b**) during potential cycling; Note that the CVs in (**a**) are from **Fig.2a** in the main text; (**c**) The maximized regions of capacitance for the CVs of YBC4 and a graphite substrate; (**d**) The evolution of integrated charge (between 1.15V and 1.55V vs. RHE) from the three electrodes during potential cycling.

As shown in **Fig. S19c**, a slight capacitance increase can be observed, and a similar trend can also be found over the substrate (a graphite paper loaded with only acetylene black, bottom of **Fig. S19c**) during the potential cycling. It has been reported that the electrochemical oxidation of the substrate, for example, the formation of surface oxidized carbon species, is responsible for this capacitance increase.[17] As to the BSCF electrode (**Fig. S19b**), its activity steeply increases at the initial 100 cycles of potential cycling. Such activity maintains in the following 900 cycles. This activity evolution for the BSCF electrode has been well studied and is related to the reconstruction of the BSCF surface.[9] That is the formed certain Co/Fe oxyhydroxides, after surface Ba/Sr leaching, are responsible for the measured activity during potential cycling. This conclusion is also demonstrated by the greatly increased capacitance, which shows distinctive Co^{x+}-related redox peaks. This is different from the capacitance increase observed in the YBC4 electrode and the

substrate. In addition, as shown in **Fig. S19d**, both the substrate and the YBC4 electrode show a similar increase in the integrated charge, indicating the observed capacitance increase from the YBC4 electrode is related to the substrate. This also highlights the surface stability of YBC4 material. Nevertheless, the integrated charge from the BSCF electrode increased by ~4 times at the initial 100 cycles, and such an increase cannot be attributed merely to the substrate. The surface reconstruction should be responsible for such charge increase.

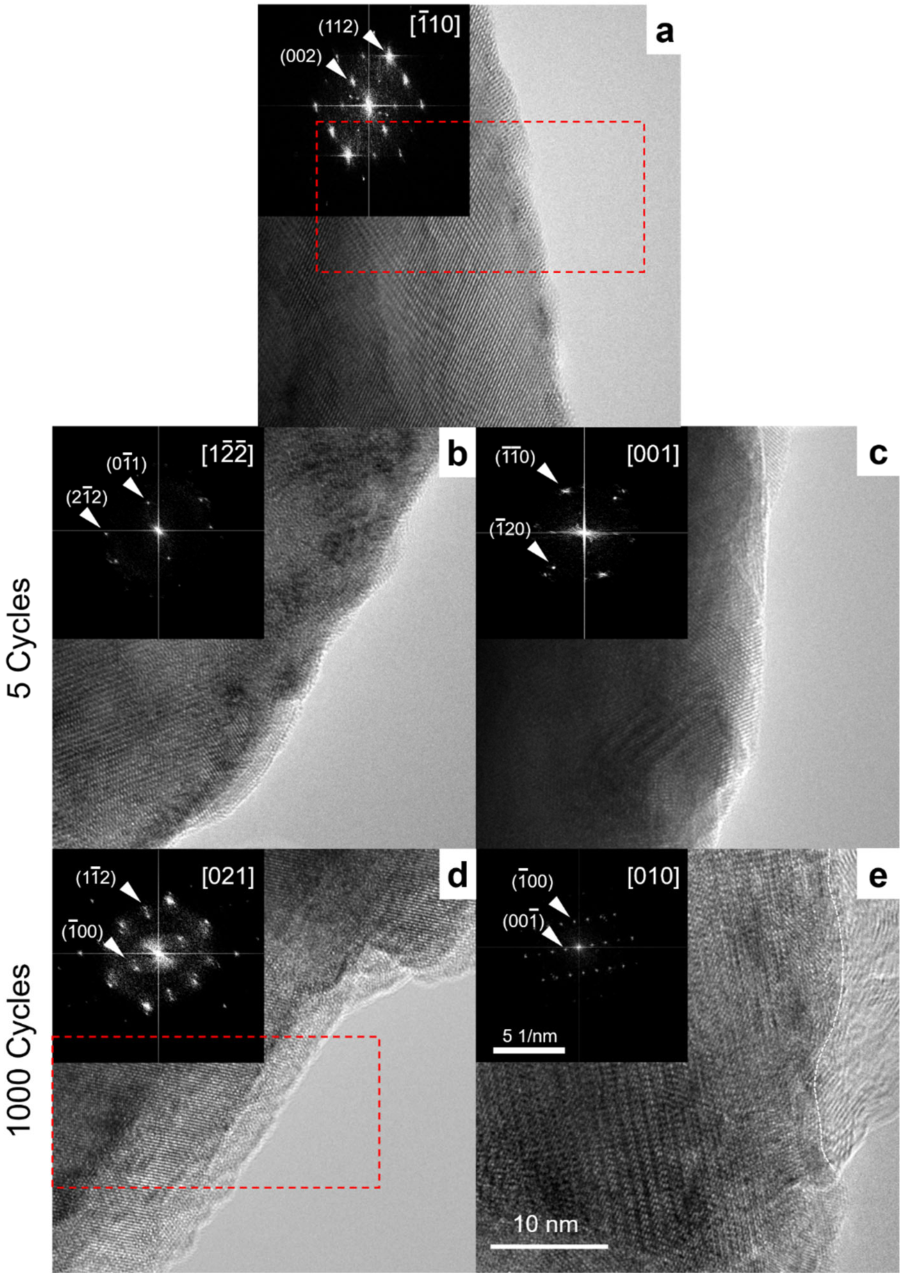

Fig. S20 TEM images of surface structure | TEM images of surface structure and corresponding FFT images from (**a**) pristine YBC4 without electrochemical test, (**b&c**) YBC4 after 5 cycles of CV scanning, and (**d&e**) YBC4 after 1000 cycles of CV scanning. A space group of P *6₃mc* is applied for indexing the FFT images. In (**a&d**), the marked regions are shown in **Fig. 3a** of the main text. In (**e**), the surface is partially overlapped with the conductive carbon. A white dash curve is used to mark the boundary. The surface of cycled YBC4 is well crystallized.

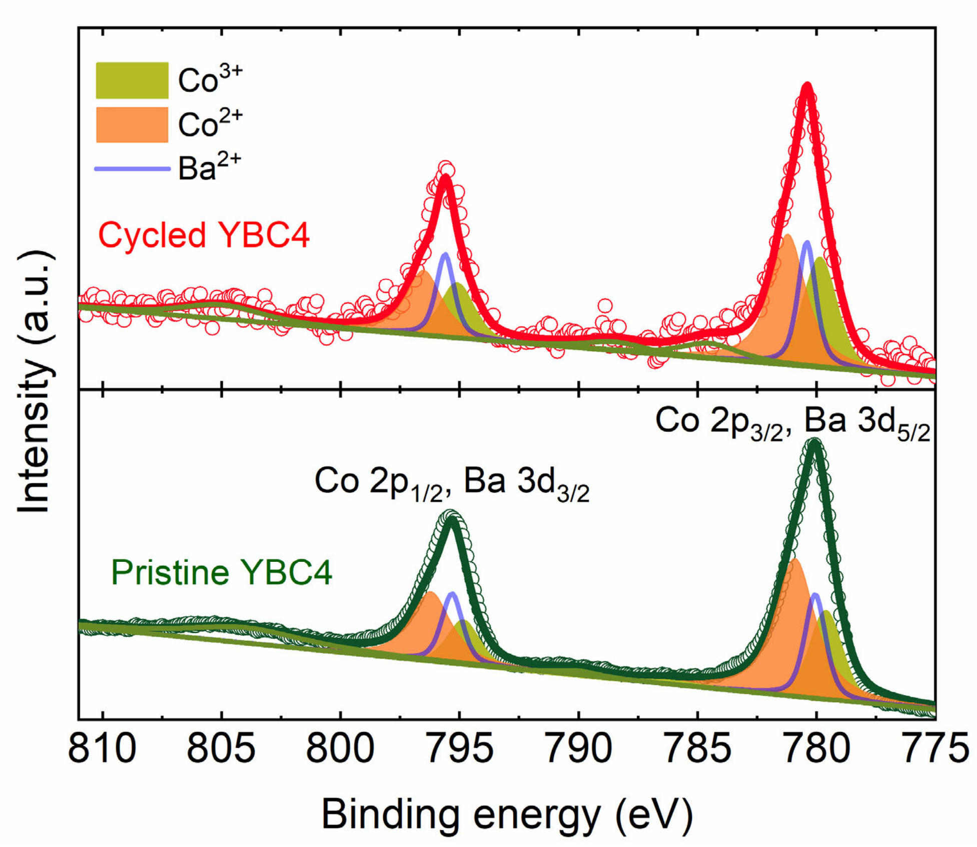

Fig. S21 Fitted Co 2p core spectra | Fitted Co 2p core spectra from the pristine YBC4 and the cycled YBC4. The spectrum of the cycled YBC4 sample is like the one from the pristine YBC4 sample. The peaks from the two samples can be fitted with three doublets, which correspond to Co^{2+}, Co^{3+}, and Ba^{2+}. The fitting results are listed in **Table S4**. Based on the fitting area from Co^{2+} and Co^{3+}, an averaged cobalt oxidation state of ~2.3+ is then estimated for the pristine YBC4, which is consistent with the XANES analysis (**Fig. S3**). Slight oxidation of surface cobalt with an averaged oxidation state of ~2.4+ is found for the cycled YBC4, indicating the electrochemical oxidation of YBC4 during OER.

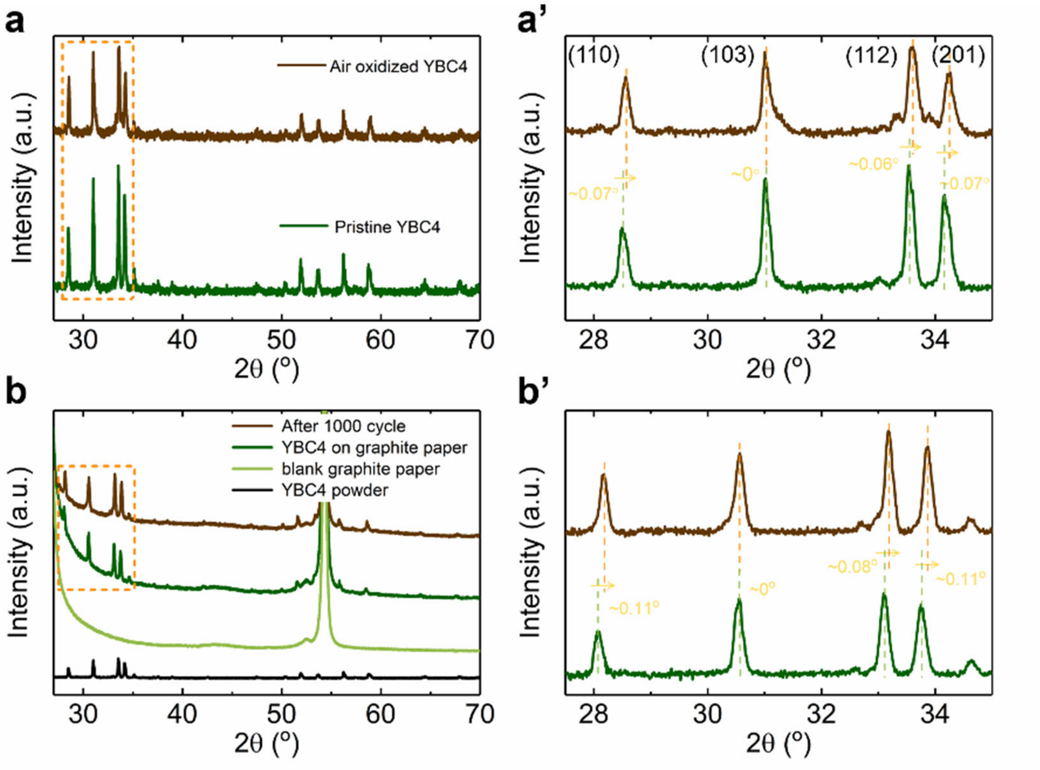

Fig. S22 XRD patterns of YBC4 before and after thermal/electrochemical oxidation | (**a&a'**) The XRD patterns of YBC4 before and after thermal oxidation. The thermally oxidized YBC4 is prepared by calcining the pristine YBC4 at 320 ºC in ambient air for 2h. (**b&b'**) The XRD patterns of YBC4 before and after 1000 cycles of potential cycling. The patterns from as-prepared YBC4 (YBC4 powder) and the electrode substrate of graphite paper are also presented as references.

In general, three factors can induce the shift of XRD peaks. The first one is the sample displacement during the XRD measurement. To avoid this, the XRD patterns for all samples are calibrated with the diffraction peaks of the graphite paper substrate (**Fig. S22b**). The second one is the thermal treatment, which may cause a lattice expansion/contraction. This is unlikely in our work as all tests are performed at room temperature. The last one is the sample oxidation, including changes in ionic size (the Co^{x+} in YBC4) and atom rearrangements (tilting, rotating, and coordination change of CoO4 tetrahedra in YBC4), which then causes the lattice change. As a result, we believe that the changed lattice parameter is due to the electrochemical oxidation of YBC4, i.e., oxygen uptake. Moreover, it is found that the XRD patterns from YBC4 after thermal oxidation (**Fig. S22a&a'**) and electrochemical cycling (**Fig. S22b&b'**) have a similar trend of peak shift. In detail, As shown in **Fig. S22a'** and **Fig. S22b'**, in both cases, the peaks belonging to

(110), (112), and (201) planes shift right while the peaks belonging to (103) plane show no shift. Such a similar trend of peak shift indicates the behavior of oxygen uptake during electrochemical cycling is like the one due to thermal oxidation.

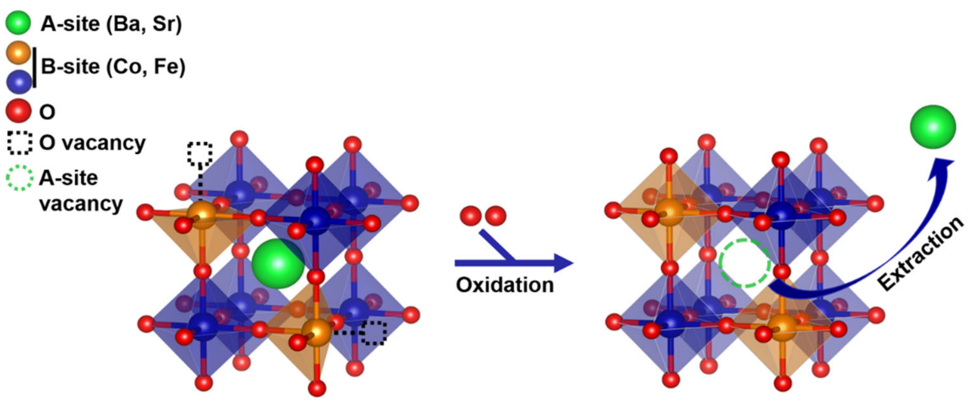

Fig. S23 Oxidation of perovskite | The oxidation of the perovskite ($ABO_{3-\delta}$) with corner-shared BO6 octahedra. Generally, the A-site is occupied by alkaline-earth-metal, such as Ba and Sr, and the B-site is occupied by transition metals, such as Co and Fe. The oxygen vacancy (oxygen nonstoichiometry of δ) may exist in the oxygen site for charge neutrality of the perovskite.

During the oxygen evolution process at high overpotential (electrochemical oxidation occurs), the oxygen vacancies in the lattice of perovskite-type materials will be filled and the transition metal ions in the B-site will be oxidized to a higher valence state, which has been proposed as an origin of the high activity in previous studies.[18,19] However, to maintain an ideal perovskite structure with fixed A-site and B-site positions, the average ionic size between A-site cations (r_A) and B-site cations (r_B) should be well matched and can be judged by a Goldschmidt tolerance factor (t), which can be calculated according to the equation of $t = \frac{r_A + r_O}{\sqrt{2}(r_B + r_O)}$. A t value close to 1 is necessary for obtaining an ideal cubic perovskite structure. Then, a greatly reduced ionic size of these transition-metal ions at a high valence state causes a large size mismatch, which will produce elastic energy within the lattice if the lattice keeps a cubic structure. Such elastic energy will force the extraction of larger A-site cations, such as Ba/Sr^{2+}, to the surface.[20] This behavior will also promote the dissolution of A-site cation during the OER process. For example, a large cation size mismatch exists in an ideal cubic BSCF phase at room temperature as the cubic structure is thermodynamically unstable and will slowly collapse to a hexagonal phase with face-shared BO6 octahedra.[21,22] additionally, it was found that the structural amorphization in BSCF only happened at a potential higher than ~1.5 V vs. RHE, i.e., with a more oxidizing condition, the original corner-shared BO6 octahedra will change to edge-shared BO6 octahedra in the formed amorphous surface region.[9] This phenomenon further signifies that the cation extraction, caused by ionic size mismatch, can contribute to this surface amorphization.

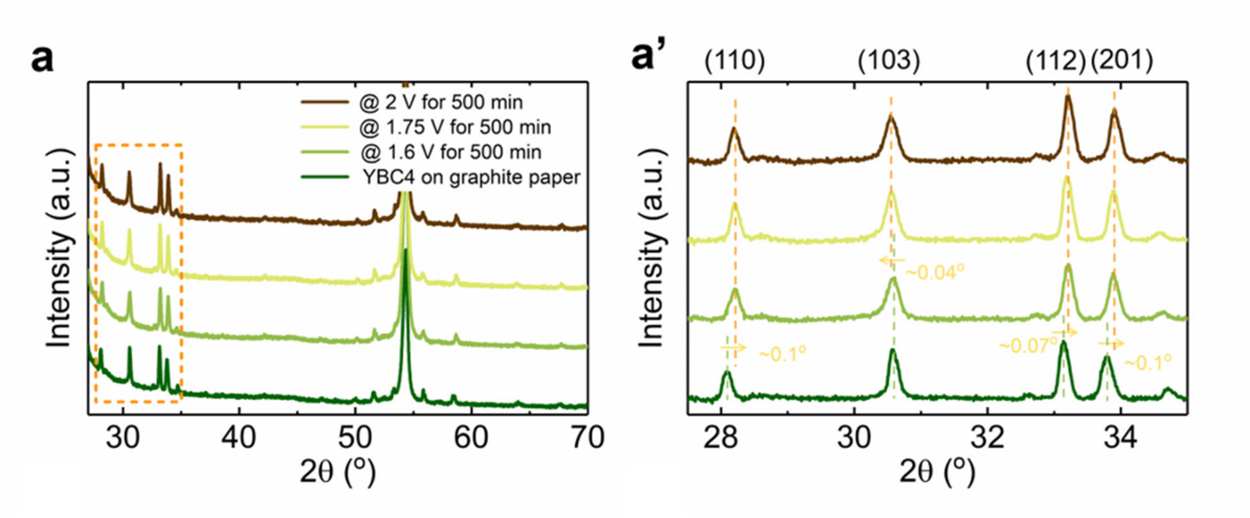

Fig. S24 XRD patterns of YBC4 before and after electrochemical oxidation | (**a&a'**) The XRD patterns of YBC4 before and after electrochemical oxidation at 1.6 V, 1.75 V, and 2 V (vs. RHE).

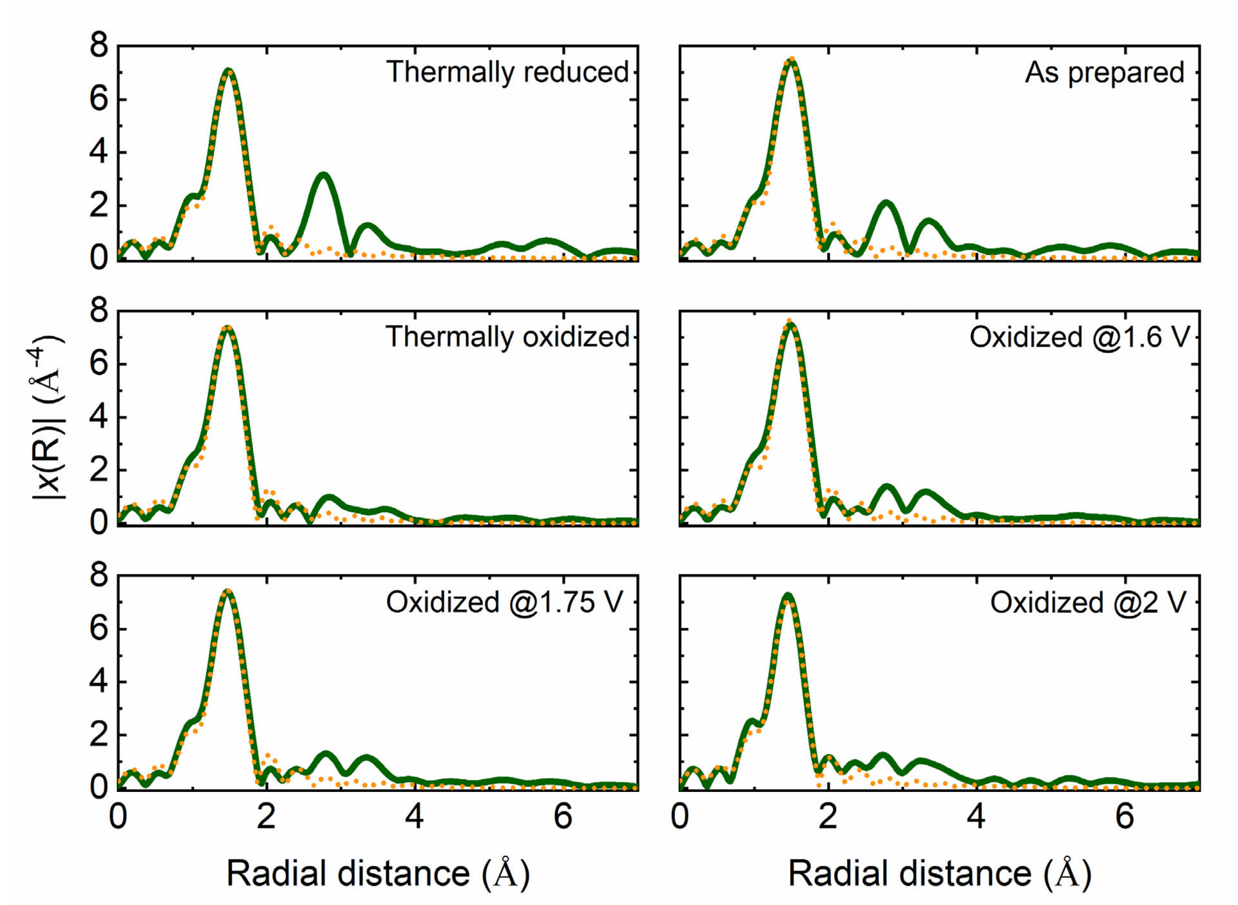

Fig. S25 Co K-edge EXAFS spectra of different YBC4 samples | k^3-weighted Co K-edge EXAFS spectra of as prepared, thermally reduced, thermally oxidized, and electrochemically oxidized (@1.6V, 1.7V, and 2V vs. RHE) YBC4. The thermally reduced YBC4 is prepared by calcining the as-prepared YBC4 at 450 ºC in Ar for 2h. The thermally oxidized YBC4 is prepared by calcining the pristine YBC4 at 320 ºC in ambient air for 2h.

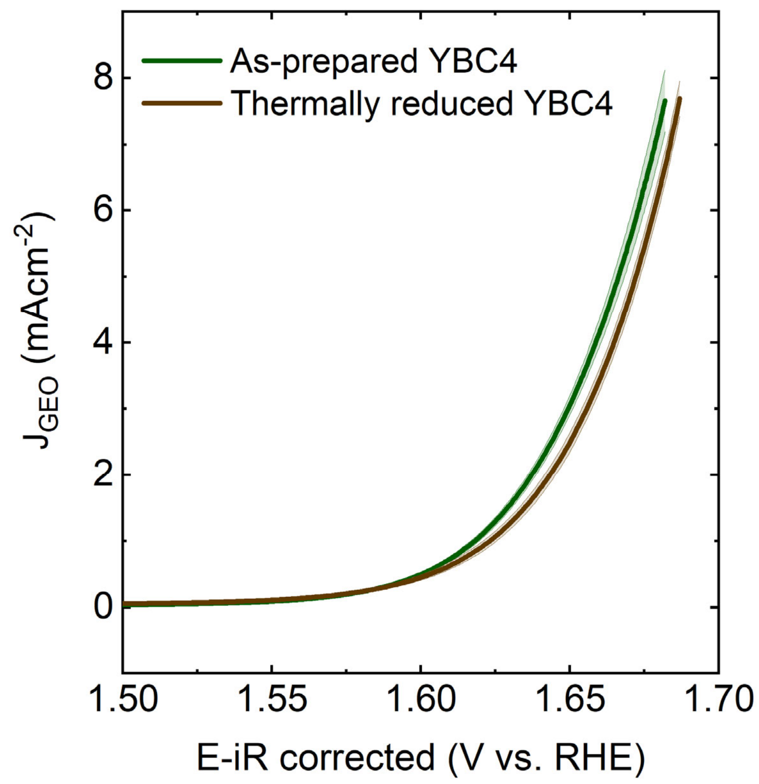

Fig. S26 OER currents for as-prepared YBC4 and Thermally reduced YBC4 | iR-corrected and electrode surface area normalized OER currents for as-prepared YBC4 and thermally reduced YBC4.

According to the reported thermogravimetric test of YBC4, the YBC4 will lose all interstitial oxygen by heating to above ~ 400 °C.[23] Thus, the formation of strictly $YBCo_4O_7$ is available with additional heat treatment. A thermally reduced YBC4 has been synthesized by treating the as-prepared YBC4 in flow Ar at 450 °C for 2h. The oxygen stoichiometry in this thermally reduced YBC4 is ~7. As shown in **Fig. S26**, the OER currents of thermally reduced YBC4 and as-prepared YBC4 are nearly identical, signifying that the minor Co octahedra in the as-prepared YBC4 cannot be the origin of the measured catalytic activity.

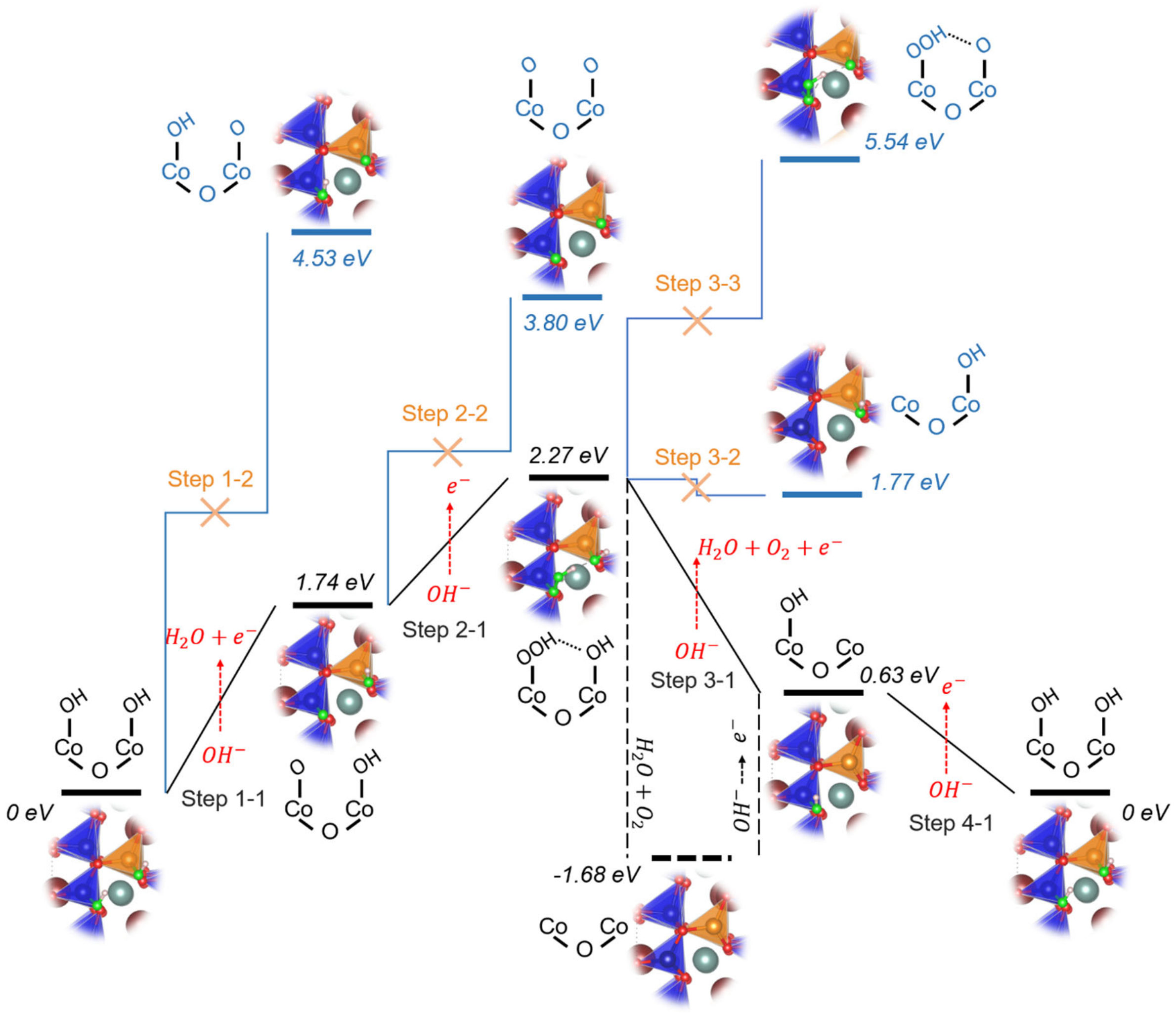

Fig. S27 Calculated possible paths in the OER on the YBC4 (110) surface | The calculated possible paths in the OER on the YBC4 (110) surface. The free energies of different possible surface statuses in each proton-coupled electron transfer step are also presented. The bonded oxygens from OER intermediates are highlighted in green.

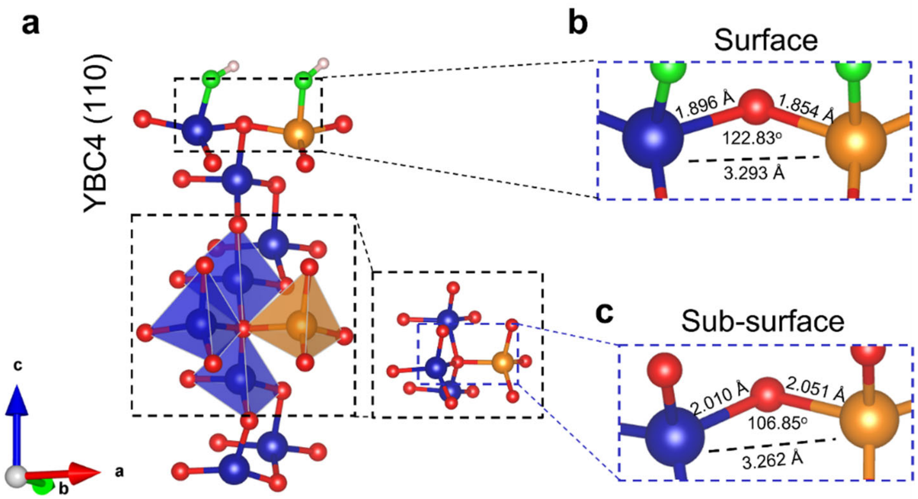

Fig. S28 Local structures in YBC4 **|** (**a**) The optimized YBC4 (110) facet with the OH-adsorbed active site; (**b**) the local structure of OH-adsorbed surface active site; (**c**) the local structure of a motif, resembles the surface active site, extracted from the sun-surface of YBC4. As compared with the structural parameters in the sub-surface tetrahedra, the Co-O bond length is shorter, and the Co-O-Co bond angle is larger in the surface-active site. This surface structural self-regulation can be explained by the character of corner-shared tetrahedra, which are easier of deformation and rotation.[24,25]

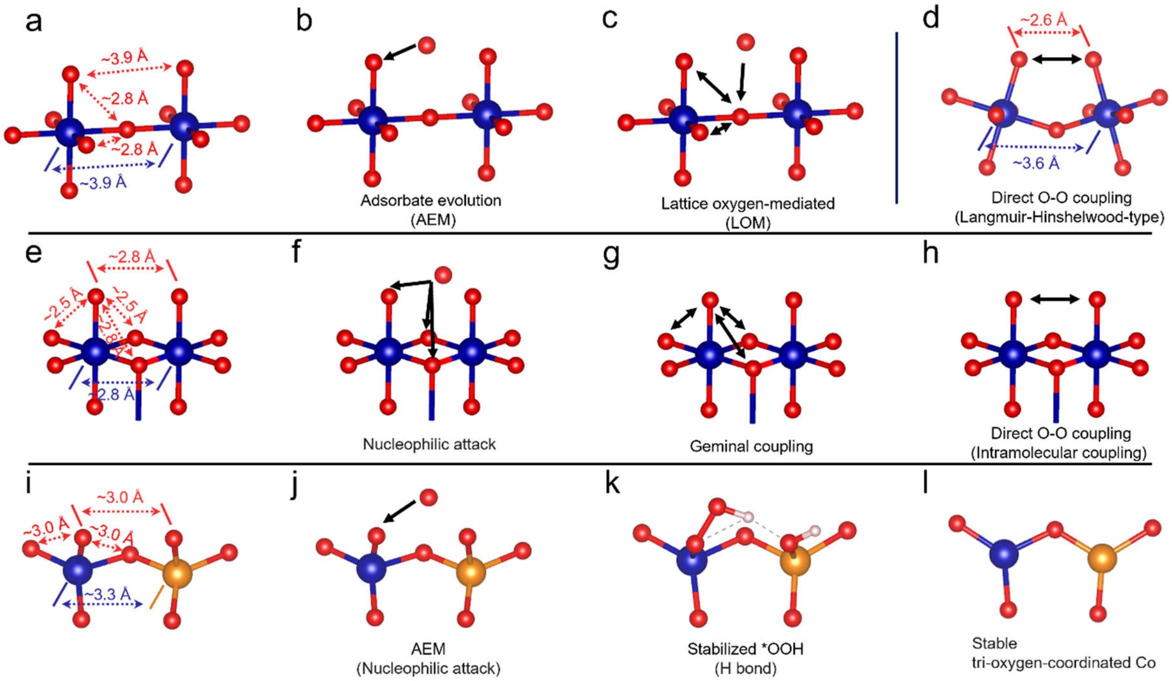

Fig. S29 Interatomic distances | Corner-shared CoO6 (**a**), edge-shared CoO6 (**e**), and corner-shared CoO4 (**i**). The possible O-O bond formation steps in corner-shared (**b-d**) and edge-shared MO6 octahedra (**f-h**). The key intermediate steps (**j-l**) in the proposed OER mechanism from dual corner-shared cobalt tetrahedra.

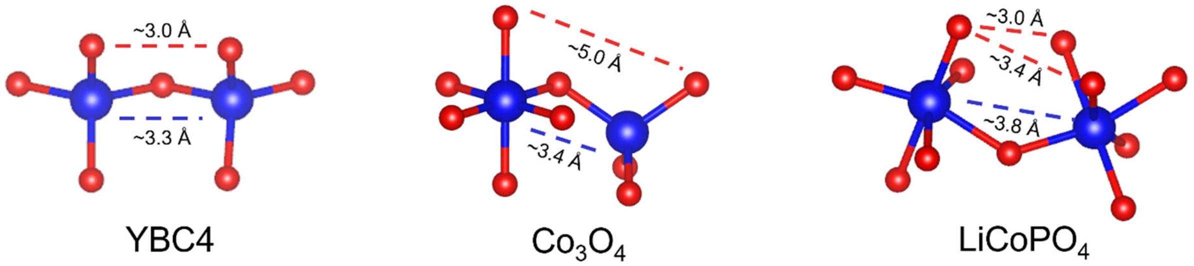

Fig. S30 Another two mono-μ-oxo-bridged Co | The mono-μ-oxo-bridged Co from YBC4, spinel of Co_3O_4, and olivine of $LiCoPO_4$.

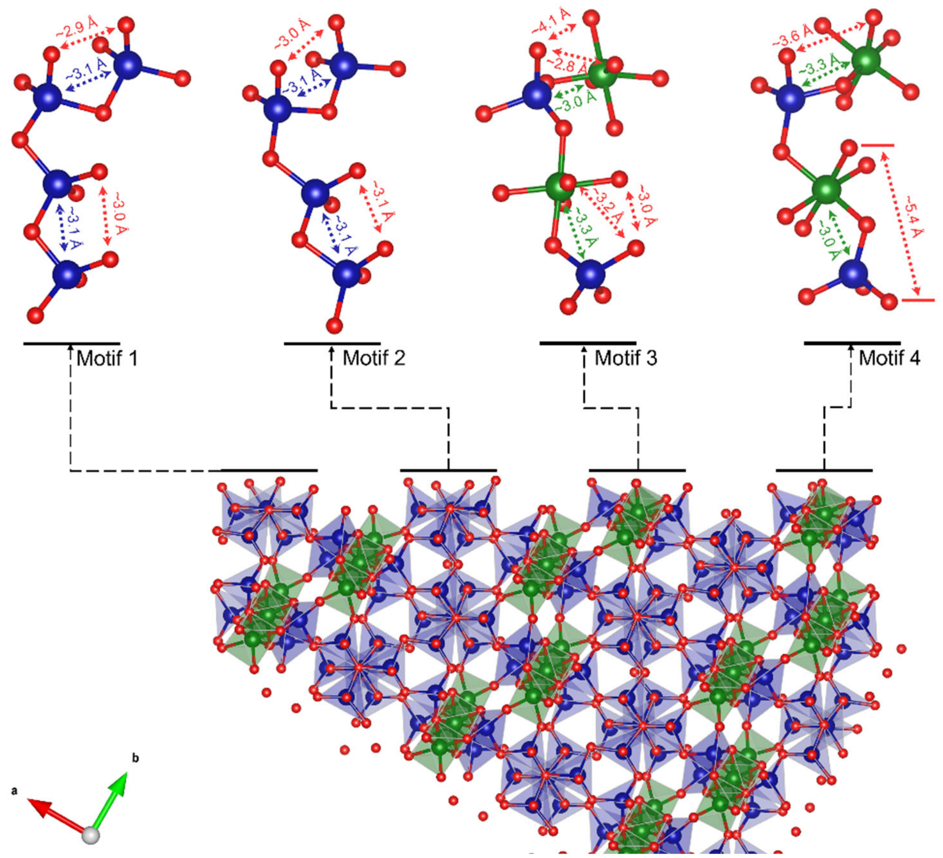

Fig. S31 The formation of "tri-oxygen-coordinated cobalt" | The bottom is the side view of the likely (110) surface structure of YBC4 after electrochemical oxidation. The top is the top view of different active motifs. The adjacent Co-Co and surface O-O distances are marked.

Supplementary Tables

Table S1 Refined structure information.

	YBC4
Space group	P 6_3mc
a(Å)	6.2882(1)
c(Å)	10.249(7)
	Y
Wyckoff	2b
site	(2/3, 1/3, 0.872(4))
Occ.	1
U_{iso} (Å^2)	0.089
	Ba
Wyckoff	2b
site	(2/3, 1/3, 1/2)
Occ.	1
U_{iso} (Å^2)	0.0275
	Co1
Wyckoff	2a
site	(0, 0, 0.426(7))
Occ.	1
U_{iso} (Å^2)	0.025
	Co2
Wyckoff	6c
site	(0.167(6),0.832(3),0.688(5))
Occ.	1
U_{iso} (Å^2)	0.027
	O1
Wyckoff	6c
site	(0.51(5), 0.48(4), 0.72(8))
Occ.	1
U_{iso} (Å^2)	0.0044
	O2
Wyckoff	2a
site	(0,0,0.26(1))
Occ.	1
U_{iso} (Å^2)	0.14
	O3
Wyckoff	6c
site	(0.15(0), 0.84(9),0.50(0))
Occ.	1
U_{iso} (Å^2)	0.014
X^2	1.358
R_p	1.42%
R_{wp}	1.89%

Table S2 Detailed parameters from XPS fitting of O 1s, Y 3d, and Ba 4d.

	Species	BE(eV)	FWHM (eV)	Area
RT	O 1s-H_2O	533.2	1.56	3441.7
	O 1s-CO_3	531.8	1.63	18154.1
	O 1s-OH	531.1	1.47	12980.7
	O 1s-lattice surface	530.1	1.71	12980.7
	O 1s-lattice bulk	529.3	1.45	7495.5
	Y 3d 3/2-O	158.8	1.60	2101.3
	Y 3d 5/2-O	156.4	1.43	3151.9
	Y 3d 3/2-CO_3	159.8	1.62	2475.2
	Y 3d 5/2-CO_3	157.7	1.55	3712.8
	Ba 4d 3/2-O	91.9	2.01	7894.3
	Ba 4d 5/2-O	89.3	1.79	11841.5
300 ºC	O 1s-H_2O	533.1	1.21	1660.3
	O 1s-CO_3	531.9	1.48	11755
	O 1s-OH	531.0	1.45	6402.6
	O 1s-lattice surface	530.0	1.62	14713.5
	O 1s-lattice bulk	529.2	16966	16966
	Y 3d 3/2-O	158.6	1.42	3032.7
	Y 3d 5/2-O	156.3	1.37	4549.1
	Y 3d 3/2-CO_3	159.6	1.76	2882.4
	Y 3d 5/2-CO_3	157.5	1.83	4323.5
	Ba 4d 3/2-O	92.0	2.23	9627.0
	Ba 4d 5/2-O	89.4	2.10	14440.6
500 ºC	O 1s-H_2O	/	/	/
	O 1s-CO_3	532.0	1.74	9070
	O 1s-OH	531.1	1.49	7874
	O 1s-lattice surface	530.1	1.56	18161.6
	O 1s-lattice bulk	529.4	1.48	19509
	Y 3d 3/2-O	158.7	1.60	4456.8
	Y 3d 5/2-O	156.5	1.48	6685.1
	Y 3d 3/2-CO_3	159.7	1.87	1789.0
	Y 3d 5/2-CO_3	157.7	1.71	2683.5
	Ba 4d 3/2-O	92.1	2.16	12263.5
	Ba 4d 5/2-O	89.4	2.10	18395.2

Table S3 ICP-MS results from used electrolytes after potential cycling.

	Y (ppb)	Ba (ppb)	Sr (ppb)
Blank electrolyte	0.102	0.002	0.023
YBC4	0.040	N/A*	0.917^
BSCF	0.0203	729	608

*The concentration of Ba is below the detection limit.

^The more Sr leaching (compared with blank electrolyte) is related to the substrate and/or glassware.

Table S4 Detailed parameters from XPS fitting of Co 2p.

	Species	BE(eV)	FWHM (eV)	Area
Pristine YBC4	Co^{3+} 2p 3/2	779.6	1.6	18527.8
	Co^{3+} 2p 1/2	794.8	1.6	9263.9
	Co^{2+} 2p 3/2	780.9	2.0	43738.4
	Co^{2+} 2p 1/2	796.2	2.0	21869.2
	Ba 3d 5/2	780.1	1.05	15420.6
	Ba 3d 3/2	795.3	1.05	10280.4
	Satellite1	786	2	682.9
	Satellite2	790	2	1328.3
	Satellite3	804	6	14575.4
Cycled YBC4	Co^{3+} 2p 3/2	779.9	1.7	1648.1
	Co^{3+} 2p 1/2	795.1	1.7	824.1
	Co^{2+} 2p 3/2	781.2	1.8	2496.6
	Co^{2+} 2p 1/2	796.5	1.8	1248.3
	Ba 3d 5/2	780.4	0.9	1102.7
	Ba 3d 3/2	795.6	0.9	735.1
	Satellite1	784.5	3	784.5
	Satellite2	788.5	3	788.5
	Satellite3	805	4	805

Table S5 Fitting parameters of the Fourier-transformed k^3-weighted Co K-edge EXAFS from YBC4 samples.

	**Co-O (Å)**	**CN#**	**σ^2 ($Å^2$)^**	**ΔE_0***	**R-factor**
YBC4-thermally reduced	1.917(0.011)	4.00(0.46)	0.0044(0.0014)	0.35(1.47)	0.0079
YBC4-as prepared	1.918(0.012)	4.08(0.51)	0.0039(0.0017)	0.42(1.60)	0.0105
YBC4-thermally oxidized	1.900(0.011)	4.24(0.52)	0.0046(0.0016)	0.12(1.58)	0.0092
YBC4-oxidized@1.6V	1.913(0.013)	4.27(0.53)	0.0041(0.0017)	0.03(1.61)	0.0106
YBC4-oxidized@1.75V	1.908(0.012)	4.29(0.50)	0.0045(0.0017)	-0.36(1.50)	0.0088
YBC4-oxidized@2V	1.903(0.013)	4.28(0.53)	0.0050(0.0018)	-0.78(1.63)	0.0114

CN: Coordination number
^ **σ^2:** Mean-square-displacement in R
* **ΔE_0**: Energy shift

Table S6 Estimated values of key parameters from YBC4, $SrCoO_3$, and CoOOH.

	Corner-shared CoO4 (YBC4)	Corner-shared CoO6 ($SrCoO_3$)	Edge-shared CoO6 (CoOOH)
d electrons (Outer electrons)	~6.7	~5	6
Charge-transfer energy (eV)	6.72	4.49 (4.64 for BSCF)	7.16
e_g/t_2 electrons	3	0~2	0~2
M-O-M bond angle (°)	~107	180	~96
Average M-O bond length (Å)	1.980(Tri) 1.887(Kagome)	1.921	1.915
M-M distance (Å)	3.293	3.842	2.851

Supplementary Discussion

A detailed comparison between Co tetrahedra and octahedra

Note that the origins of activity of octa-coordinated metal have been well explored. Several key parameters/descriptors of the octa-coordinated metal (MO6), including d-band electron number, e_g electron number, charge transfer energy, metal-oxygen bond length, and metal-metal bond length, have been used in predicting the corresponding catalytic activity.[26] For a better understanding of the proposed OER mechanism over Co tetrahedra, the tetra-coordination site in YBC4 and the typical octa-coordination sites in other catalysts were carefully compared, and the effectiveness of the reported key parameters/descriptors in tetra-coordination site were evaluated.

Table S6 presents the key parameters from YBC4, $SrCoO_3$, and CoOOH. The $SrCoO_3$, composed of corner-shared CoO6, is a representative perovskite with high OER catalytic activity.[27] The CoOOH, composed of edge-shared CoO6, is a common layered oxyhydroxide with moderate OER catalytic activity.[28]

The number of d electrons

A positive correlation between the number of d electrons in transition metal (TM) ions and OER activity was first identified in a series of perovskite oxides.[29] Specifically, a higher number of d electrons means that the sigma antibonding of TM-OH can be occupied with more electrons, resulting in a weaker bond. Such a weak TM-OH bond can facilitate the desorption of OH from TM, a rate-limiting step in the studied perovskites. This concept was further developed by exploring the correlation between adsorption energies of three OER intermediates (including *OH, *O, and *OOH, * is the active TM center) and the number of outer electrons (valence electrons of TM ions).[30] The theoretical calculations reveal that the adsorption energies of all three intermediates over the active site decrease systematically as the number of outer electrons increases. The consistent adsorption-energy changes correspond well with the proposed existence of adsorption-energy scaling relations among OER intermediates.[31] Such scaling relation, on the other hand, has been found useful in predicting superior catalysts with "optimal" adsorption properties for OER.[32]

In a typical adsorption evolution mechanism, the binding energy difference between *OH and *OOH ($\Delta G_{OOH} - \Delta G_{OH}$) is about $3.2 \pm 0.2\ eV$, which is higher than the ideal value of 2.46 eV ($1.23\ eV \times 2$). On the other hand, the ($\Delta G_{OOH} - \Delta G_{OH}$) can also be expressed as ($\Delta G_{OOH} - \Delta G_{O}$)+ ($\Delta G_{o} - \Delta G_{OH}$). Then, an "optimal" OER catalyst, with the ($\Delta G_{o} - \Delta G_{OH}$) close to 1.6 eV

(3.2 $eV/2$), is expected with the lowest overpotential.[32] A ($\Delta G_O - \Delta G_{OH}$) higher or lower than 1.6 eV means too weak or too strong adsorption energy, respectively. Given that the number of d electrons (outer electrons) can greatly influence the adsorption of OER intermediates, it is reasonable that the number of d electrons has been identified as the most important activity descriptor in perovskites with the active site in an octahedral geometry.[26]

The number of d electrons, however, may not be applicable in predicting the activity of YBC4. This is because the adsorption-energy scaling relation is circumvented in YBC4. The $\Delta G_{OOH} - \Delta G_{OH}$ is calculated to be 2.27 eV, which is substantially lower than the typical value of 3.2 eV. The circumvented scaling relation in YBC4 is due to the formation of a hydrogen bond between adjacent *OOH and *OH. The details are discussed in "**Geometry structure**". The descriptor of d electron numbers can take effect only if the adsorption-energy scaling relation exists and the OER process follows the typical adsorption evolution mechanism.

Charge-transfer energy (CTE)

The CTE is defined as the energy difference between the unoccupied metal 3d- and occupied O 2p-band centers, and it reflects the metal-oxygen covalency.[33] A lower CTE indicates a higher metal-oxygen covalency, hinting at reduced oxygen vacancy formation energy and electron-transfer energy.[34] On the other hand, both the easier oxygen vacancy formation and the facilitated electron transfer in the catalyst can be beneficial for catalyzing the OER.[33,35] As a result, a high OER activity is often observed from the material (e.g., perovskites and spinels) with low CTE.

As presented in **Table S6**, the CTE of YBC4 is calculated to be 6.7 eV, which is higher than the 4.49/4.64 eV in $SrCoO_3$/BSCF and comparable with the 7.16 eV in CoOOH. The high CTE of YBC4 may be related to the CoO4 tetrahedra, in which the spatial overlap between O p-orbitals and Co d-orbitals is relatively low.[36] Thus, the measured high OER catalytic activity of YBC4 cannot be related to its metal-oxygen covalency.

The number of e_g electrons

The correlation between OER activity and the filling of the e_g orbitals of transition metal was initially proposed by Shao-Horn et al.[12] They found a highly active BSCF perovskite and proposed that the corresponding e_g occupation of close to unity can be optimal for OER catalysts. Based on the crystal field theory, in octahedral symmetry, the d-orbitals split into triply-degenerated t_{2g} orbitals (low energy) and doubly-degenerated e_g orbitals (high energy). Since the lobes of e_g

orbitals (d_{z^2} and $d_{x^2-y^2}$) point directly along the M-O bonding axes, the e_g orbitals are strongly overlapped with the O p orbitals from OER intermediates (sigma bonds). On the other hand, weaker pi bonds are formed between the t_{2g} orbitals and O p orbitals. Thus, it is reasonable to propose that the e_g occupation determines the binding between OER intermediates and oxide surface, and as a result, influences the OER catalytic activity.

In tetrahedral symmetry, the d-orbitals split into triply-degenerated t_2 orbitals (high energy) and doubly-degenerated e orbitals (low energy). The higher energy of t_2 orbitals is related to its stronger overlap with O p orbitals. However, unlike the case in octahedral symmetry, none of the five orbitals in tetrahedral geometry point directly along the M-O bonding axes. As a result, the interaction between t_2 and O p orbitals is weaker than the interaction between e_g and O p orbitals in octahedral symmetry. Due to such weak interaction, the crystal field splitting energy in tetrahedral symmetry (Δ_{tet}) is also much lower than the energy in octahedral symmetry (Δ_{oct}). As a result, a high spin state is often expected for the metal in tetrahedral symmetry. For example, the number of t_2 electrons of Co in YBC4 can always be three and, according to the oxidation state of Co, the corresponding number of e electrons may vary between 4 and 2. In addition, the relatively weak interaction between t_2 and O p orbitals hints the filling of e orbitals in a tetrahedron may also be important for the OER. Finally, the demonstrated optimal e_g occupation of close to unity in octahedral sites may not be applicable for the t_2 orbitals in tetrahedral sites.

Geometry structure

The geometry parameters of M-O-M bond angle and M-O bond length (corresponding to tolerance factor in perovskite structure) have been identified as secondary activity descriptors.[26] However, unlike the above-mentioned descriptors, the "optimal values" for these geometry parameters have not been specified. It has been proposed that the M-O-M bond angle may alter the CTE, which influences the catalytic activity. And a bond angle close to 180° is found with the lowest CTE.[37] This corresponds well with the fact that many highly active perovskites adopt a cubic or *pseudo*-cubic structure, in which the MO6 octahedra are corner-shared and M-O-M bonds are almost straight.

In the case of YBC4, the CoO4 tetrahedra are also corner-shared, but the Co-O-Co bond angle is close to 100° (~107° from bulk and ~123° from surface site). In combination with the estimated

high CTE (6.72 eV) in YBC4, the corresponding Co-O-Co bond angle cannot be an "optimal value".

Additionally, based on the foregoing discussions, the electronic structure of CoO4 in YBC4 makes a single CoO4 unlike a highly active site for OER. This corresponds well with the proposed OER process for YBC4, in which the reaction proceeds over two tetrahedra of mono-μ-oxo-bridged CoO3(OH). This structural motif should be the key to the measured high activity of YBC4.

In most of the OER active sites in an octahedral geometry, the identified structural motif is either corner-shared (mono-μ-oxo-bridged) or edge-shared (di-μ-oxo-bridged) MO6 octahedra. The possible OER mechanisms over these two structural motifs have been extensively explored. For a better understanding of the correlation between the proposed OER mechanism and geometry structure/parameters in YBC4, we further compare the geometry parameters amount different structural motifs, and the corresponding OER mechanisms are also discussed. **Fig. S29** shows the possible O-O bond formation steps, which are the key differences among reported OER mechanisms.

Corner-shared MO6 octahedra

The active motif of corner-shared MO6 octahedra is often observed from the perovskite-type structure. **Fig. S29a** shows the interatomic distances in a typical cubic $SrCoO_3$ perovskite. The distances between adjacent Co, two terminal O, and adjacent O are ~3.9 Å, ~3.9 Å, and ~2.8 Å, respectively. Mainly three types of O-O bond formation mechanisms have been proposed based on perovskites. The first is the general adsorbate evolution mechanism (AEM), the second is the lattice oxygen-mediated mechanism (LOM), and the third is the direct O-O coupling mechanism.[11,12,27,35,38]

As shown in **Fig. S29b**, in the AEM, the O-O bond formation is related to the water nucleophilic attack, which proceeds on an adsorbed oxygen (terminal oxygen).[12,38] In the LOM, the lattice oxygen is involved in the O-O bond formation.[27,35] As shown in **Fig. S29c**, the O-O bond can form from the coupling of one lattice oxygen and one adsorbed oxygen, the coupling of adjacent lattice oxygen, and the water nucleophilic attack on lattice oxygen. The third mechanism includes the direct coupling of two adsorbed oxygen atoms (**Fig. S29d**).[11] Realization of such coupling is due to the reduced distance (~2.6 Å) between two adsorbed oxygen atoms, which is caused by the

titling of two MO6 octahedra. Different from the coupling of adjacent oxygen atoms in LOM, the lattice oxygen is not involved in the direct coupling mechanism and the adsorbed oxygen atoms are from adjacent metal centers.

Edge-shared MO6 octahedra

The edge-shared MO6 octahedra have been widely observed in state-of-the-art catalysts, such as (oxy)hydroxides, layered oxides, and cobalt catalyst films from electrodeposition.[39-42] **Fig. S29e** shows typical edge-shared MO6 octahedra from CoOOH, in which the two Co ions are interconnected by di-$\mu_{2,3}$-O. The distances between adjacent Co, two terminal O, and adjacent O are ~2.9 Å, ~2.8 Å, and ~2.8 or ~2.5 Å (distorted octahedron), respectively. Most of O-O bond formation mechanisms over edge-shared MO6 octahedra fall into three categories: water nucleophilic attack, germinal coupling, and direct O-O coupling (intramolecular coupling).

As shown in **Fig. S29f**, the nucleophilic attack can happen on either terminal oxygen or bridging oxygen.[43-46] The germinal oxygen coupling is from the coupling of the terminal and bridging oxygen atoms bonded to the same M center (**Fig. S29g**).[46,47] Note that, if we define the bridging oxygen as a kind of lattice oxygen, the bridging oxygen involved O-O bond formation in nucleophilic attack and geminal coupling mechanisms can also be considered as the LOM in corner-shared MO6 octahedra (**Fig. S29c**).[48] The direct O-O coupling is defined as the coupling of two terminal oxygen atoms (**Fig. S29h**), which is like the direct O-O coupling in corner-shared MO6 octahedra.

Edge-shared MO4 tetrahedra

Fig. S29i shows the edge-shared MO4 tetrahedra from YBC4. The distances between adjacent Co, two terminal O, and adjacent O are ~3.3 Å, ~3.0 Å, and ~3.0 Å, respectively. The correlation between the proposed OER mechanism and the edge-shared CoO4 tetrahedra over the YBC4 (110) surface can be expressed as follows:

i) OER with adsorption evolution mechanism (**Fig. S29j**)

The other two O-O bond formation mechanisms, including the coupling of adjacent O atoms (lattice oxygen mediated) and the direct coupling of two terminal O atoms, can be unlikely for several factors:

For the coupling of adjacent O atoms: 1) Lattice oxygen evolution from CoO4 is difficult. The much weaker interaction between Co d and O p orbitals in tetrahedral geometry makes the lattice oxygen from YBC4 “inert” towards OER. 2) The interatomic distance of adjacent O atoms (~3.0 Å) is relatively large. In tetrahedral geometry, the legends (O) have more free space (compared to octahedral geometry), and thus are more separated for stabilization.

For the direct coupling of two terminal O atoms: The distance between two terminal O from adjacent CoO4 tetrahedra (~3.0 Å) is relatively large. According to a reported correlation between the free energy barrier for the O-O bond formation and the interatomic distance of two terminal oxygen atoms, the two terminal oxygen atoms with an interatomic distance of ~3.0 Å are fairly stable and are not inclined to direct coupling.[49] Note that the above-mentioned correlation is estimated based on the motif of edge-shared MO6 octahedra and the correlation in edge-shared MO4 tetrahedra may change to some extent. In addition, it is known that the corner-shared tetrahedral units have high rotational flexibility.[50] It suggests that, although the direct coupling of two terminal O atoms is unlikely in YBC4, the direct O-O coupling is possible if the tetrahedral units in other materials can rotate sufficiently, which warrants further investigation.

ii) Circumvented scaling relation over the YBC4 (110) surface (**Fig. S29k**)

In brief, the scaling relation in OER is expected to be circumvented mainly in two conditions. Firstly, the OER intermediate of *OOH (* is the active site) is selectively stabilized over *OH. Generally, three strategies, including the introduction of a second active site, the addition of a proton acceptor, and the construction of a confined space, have been demonstrated in stabilizing the *OOH.[51-53] A second active site is a case where the OER intermediates are adsorbed on different active sites, a proton acceptor is a second site that can accept the protons from the OER intermediates, and a confined space is a confined reaction environment that enables selective interaction between the different reaction intermediates and the catalyst. Secondly, the formation of *OOH is circumvented. Such OER mechanism can be realized by either lattice oxygen evolution or the direct coupling of two terminal O from adjacent active sites.[35,49]

In the case of OER over the YBC4 (110) surface, the formation of a hydrogen bond (between $\overset{\triangle}{_t}* OH$ and $\overset{\triangle}{_k}* OOH$) over the dual corner-shared CoO4 additionally stabilizes the *OOH and *OH co-adsorbed YBC4 (110) surface, and thus the scaling relation is circumvented. The short adjacent Co-Co distance (<3.3 Å) allows the interaction of intermediates from two Co centers, i.e., the

formation of a hydrogen bond. Note that the Co-Co distance is not the only factor that determines the formation of hydrogen bonds. Firstly, the adsorbed intermediates, such as OOH, can self-rotate and/or -tilt to ensure the formation of a proper hydrogen bond to accommodate the “destabilization effect” from the change of Co-Co distance. Secondly, the arrangement/type of adjacent Co polyhedra would affect the possible interaction of adsorbed intermediates (please refer to the Additional discussion of another two mono-μ-oxo-bridged Co, **Fig. S30**).

The key active motif of dual corner shared CoO4 is further evaluated by checking the adsorption of OER intermediates over another possible motif of a single CoO4. Specifically, the binding energies of OER intermediates on a single tri-coordinated Co (a single CoO4 from a zinc blende CoO (111) surface) are also calculated. Values of 1.49 eV, 4.24 eV, and 4.57 eV are calculated for *OH, *O, and *OOH, respectively. The corresponding energy difference between *OH and *OOH is 3.08 eV (4.57 eV - 1.49 eV), which is within the range of the reported universal energy difference of 3.2 ± 0.2 eV. Thus, it can be concluded that the adsorption of OER intermediates, over a single CoO4, follows the scaling relation.

iii) tri-oxygen-coordinated Co (**Fig. S29l**)

The stable tri-oxygen-coordinated Co site ($\overset{\triangle}{k}* \cdots \overset{\triangle}{t}*$) over YBC4 (110) surface facilitates the coupled desorption of O_2 (from $\overset{\triangle}{k}*\ OO$) and H_2O (from $\overset{\triangle}{t}*\ OH_2$).

Additional discussion of another two mono-μ-oxo-bridged Co

Fig. S30 further compares the motifs of mono-μ-oxo-bridged Co from YBC4, spinel of Co_3O_4, and olivine of $LiCoPO_4$. The interatomic distances between adjacent metal centers and between two terminal oxygen atoms are marked. In Co_3O_4, the motif is constructed by corner-shared octahedra and tetrahedra. The Co-Co distance of ~3.4 Å is close to the value in YBC4 while the O-O distance can reach ~5.0 Å, which makes the interactions of OER intermediates from two metal centers unlikely. Furthermore, the Co_3O_4 is demonstrated with reversible phase transition during OER.[54,55] That is the tetrahedra in spinel can transform to octahedra, which induces the formation of di-μ-oxo-bridged Co (edge-shared CoO6). In such motif, the OER catalyzed by two Co metal centers has been found possible.[44]

In $LiCoPO_4$, the motif is constructed by corner-shared octahedra. The corresponding Co-Co distance is ~3.8 Å, which is close to the value (~3.9 Å) in perovskite. Due to the high degree of tilting and rotating of the two octahedra, the O-O distance can be between ~3.0 Å and ~3.4 Å. The active site of two metal centers is possible in such a motif with short O-O distance. However, the rotating of octahedra makes the bonded OER intermediates in different directions (non-cofacial), which may increase the kinetic barrier of OER.[56] On the other hand, the stability of this active site is a problem as the $LiCoPO_4$ becomes amorphous during OER. The leaching of P and/or Li likely is the cause of this instability.[57]

The effect of electrochemical oxidation

The coordination number of some Co should increase to six due to the electrochemical oxidation with additional oxygen intercalation, which gives some octahedrally coordinated Co. The formed Co octahedra may not transfer to "tri-oxygen-coordinated cobalt" during OER, and as a result, the amount of surface "tri-oxygen-coordinated cobalt" will reduce. Note that the proportion of formed Co octahedra in the oxidized YBC4 can be rather low, and Co tetrahedra are still dominant. The formation of "tri-oxygen-coordinated cobalt" from other Co sites is not obstructed by the excess oxygen. The details are discussed as follows:

The proportion of Co octahedra

Based on the EXAFS analysis of the samples after electrochemical oxidation, the mean coordination number of Co is found to be ~ 4.29, hinting that the proportion of Co octahedra in electrochemically oxidized YBC4 samples can reach ~ 15%. Moreover, due to the bulk migration/diffusion of intercalated oxygen atoms, this electrochemical oxidation-induced coordination increment should occur throughout the bulk, not only on the outer surface. This is demonstrated by the unified peak shifts in the XRD patterns (**Fig. S22&24**), which reflect the structure change of both bulk and surface. In addition, the surface TEM images after electrochemical cycling can still be well indexed based on a hexagonal phase of $YBaCo_4O_7$, indicating that the surface oxygen nonstoichiometry cannot be high (**Fig. S20**). The surface structure can be highly distorted if excess oxygen is accumulated in the surface region. For example, the structure may change from hexagonal to orthorhombic as the oxygen stoichiometry increases from 7 to 8.1.[58]

The formation of "tri-oxygen-coordinated cobalt"

The conclusion that the excess oxygen (or the formed octahedra) does not interfere with the formation of "tri-oxygen-coordinated cobalt" can be obtained from two aspects.

Firstly, the structural motif of the active site in the oxidized sample resembles the case in stoichiometric $YBaCo_4O_7$. Given that the structure of highly oxidized YBC4 has been well explored (as shown in **Fig. S31**), a possible (110) surface structure of YBC4 after electrochemical oxidation is derived from a $YBaCo_4O_{8.1}$ with an orthorhombic structure (P $bc2_1$).[58] And a close look at the structure of the oxidized surface should be helpful for a better understanding of the

effect of excess oxygen. In such an oxidized structure, the proportion of Co octahedra is 25%. Four structural motifs can be identified from the oxidized surface. It can be found that structural motifs 1&2 include only corner-shared Co tetrahedra and resemble the surface structural motif (**Fig. 1b**) in stoichiometric YBC4. The corresponding adjacent Co-Co (~3.1 Å) and surface O-O (~3.0 Å) distances are also close to the cases (~3.3 Å for Co-Co distance and ~3.0 Å for O-O distance) in YBC4, confirming the negligible effect of the excess oxygen. This can be explained by the fact that the flexible tetrahedra network can minimize the influence of structural distortion and the distorted structure is localized. As a result, the formation of "tri-oxygen-coordinated cobalt" in these two motifs is not affected by the excess oxygen and the OER still can be catalyzed by the proposed active site of mono-μ-oxo-bridged Co tetrahedra. The surface structural motifs 3&4 include the Co octahedra, which originate from the intercalation of excess oxygen. The adjacent Co is still mono-μ-oxo-bridged and the Co-Co distance of ~3.0/3.3 Å is also close to the cases in structural motifs 1&2. However, due to the structural distortion and the formation of octahedra, the surface O-O distance may reach ~5.4 Å and the terminal O from adjacent Co are largely non-cofacial, both of which are unfavorable for the interactions of OER intermediates from adjacent Co. In these two motifs, the formation of "tri-oxygen-coordinated cobalt" is still available from Co tetrahedra, but the OER may not be catalyzed by the active site of dual corner-shared cobalt tetrahedra. Meanwhile, it should be noted that the surface structure shown in **Fig. S31** is different from the oxidized real surface in the population of octahedra. As discussed above, in the real case the proportion of octahedra is lower (e.g., 15 %). Structural motifs 1&2 should be dominant in the oxidized real surface.

Secondly, although there is electrochemical oxidation happens on YBC4 during CV cycling, the YBC4 shows stable performance (**Fig. 2b**). The TEM and other characterizations show no remarkable change on the surface of YBC4, indicating that the active sites over the YBC4 surface are not affected by electrochemical oxidation. The stable performance suggests the formation of "tri-oxygen-coordinated cobalt" would not be obstructed by the excess oxygen.

References

1 Carta, D. *et al.* A structural and magnetic investigation of the inversion degree in ferrite nanocrystals MFe2O4 (M= Mn, Co, Ni). *The Journal of Physical Chemistry C* **113**, 8606-8615 (2009).

2 Durand, C. *et al.* Microstructure and electrical characterizations of yttrium oxide and yttrium silicate thin films deposited by pulsed liquid-injection plasma-enhanced metal-organic chemical vapor deposition. *Journal of applied physics* **96**, 1719-1729 (2004).

3 Dubbe, A., Wake, M. & Sadaoka, Y. Yttria/carbonate composite solid electrolytes for potentiometric CO_2 sensors. *Solid State Ionics* **96**, 201-208 (1997).

4 Andersson, S. L. T. & Otamiri, J. C. Surface and bulk composition of $YBa_2Cu_3O_{6+x}$ compounds studied by XPS. *Applied surface science* **45**, 1-12 (1990).

5 Doveren, H. v. & Verhoeven, J. T. XPS spectra of Ca, Sr, Ba and their oxides. *Journal of Electron Spectroscopy and Related Phenomena* **21**, 265-273 (1980).

6 Müller, P., Meffert, M., Störmer, H. & Gerthsen, D. Fast mapping of the cobalt-valence state in $Ba_{0.5}Sr_{0.5}Co_{0.8}Fe_{0.2}O_{3-d}$ by electron energy loss spectroscopy. *Microscopy and Microanalysis* **19**, 1595-1605 (2013).

7 Jung, S., McCrory, C. C., Ferrer, I. M., Peters, J. C. & Jaramillo, T. F. Benchmarking nanoparticulate metal oxide electrocatalysts for the alkaline water oxidation reaction. *Journal of Materials Chemistry A* **4**, 3068-3076 (2016).

8 Risch, M. *et al.* Structural changes of cobalt-based perovskites upon water oxidation investigated by EXAFS. *The Journal of Physical Chemistry C* **117**, 8628-8635 (2013).

9 May, K. J. *et al.* Influence of oxygen evolution during water oxidation on the surface of perovskite oxide catalysts. *The journal of physical chemistry letters* **3**, 3264-3270 (2012).

10 Lee, Y., Suntivich, J., May, K. J., Perry, E. E. & Shao-Horn, Y. Synthesis and activities of rutile IrO_2 and RuO_2 nanoparticles for oxygen evolution in acid and alkaline solutions. *The journal of physical chemistry letters* **3**, 399-404 (2012).

11 Yagi, S. *et al.* Covalency-reinforced oxygen evolution reaction catalyst. *Nature Communications* **6**, 8249, (2015).

12 Suntivich, J., May, K. J., Gasteiger, H. A., Goodenough, J. B. & Shao-Horn, Y. A perovskite oxide optimized for oxygen evolution catalysis from molecular orbital principles. *Science* **334**, 1383-1385 (2011).

13 Han, B. *et al.* Role of $LiCoO_2$ surface terminations in oxygen reduction and evolution kinetics. *The journal of physical chemistry letters* **6**, 1357-1362 (2015).

14 Wei, C. *et al.* Cations in octahedral sites: a descriptor for oxygen electrocatalysis on transition-metal spinels. *Advanced Materials* **29**, 1606800 (2017).

15 Xu, X. *et al.* Toward enhanced oxygen evolution on perovskite oxides synthesized from different approaches: a case study of $Ba_{0.5}Sr_{0.5}Co_{0.8}Fe_{0.2}O_{3-\delta}$. *Electrochimica Acta* **219**, 553-559 (2016).

16 Zhu, Y. *et al.* $SrNb_{0.1}Co_{0.7}Fe_{0.2}O_{3-\delta}$ perovskite as a next-generation electrocatalyst for oxygen evolution in alkaline solution. *Angewandte Chemie* **127**, 3969-3973 (2015).

17 Avasarala, B., Moore, R. & Haldar, P. Surface oxidation of carbon supports due to potential cycling under PEM fuel cell conditions. *Electrochimica Acta* **55**, 4765-4771 (2010).

18 Zhu, Y. *et al.* Enhancing electrocatalytic activity of perovskite oxides by tuning cation deficiency for oxygen reduction and evolution reactions. *Chemistry of Materials* **28**, 1691-1697 (2016).

19 Kim, J., Yin, X., Tsao, K.-C., Fang, S. & Yang, H. $Ca_2Mn_2O_5$ as oxygen-deficient perovskite electrocatalyst for oxygen evolution reaction. *Journal of the American Chemical Society* **136**, 14646-14649 (2014).
20 Lee, W., Han, J. W., Chen, Y., Cai, Z. & Yildiz, B. Cation size mismatch and charge interactions drive dopant segregation at the surfaces of manganite perovskites. *Journal of the American Chemical Society* **135**, 7909-7925 (2013).
21 Chen, Y. *et al.* Role of silver current collector on the operational stability of selected cobalt-containing oxide electrodes for oxygen reduction reaction. *Journal of Power Sources* **210**, 146-153 (2012).
22 Švarcová, S., Wiik, K., Tolchard, J., Bouwmeester, H. J. & Grande, T. Structural instability of cubic perovskite $Ba_xSr_{1-x}Co_{1-y}Fe_yO_{3-\delta}$. *Solid State Ionics* **178**, 1787-1791 (2008).
23 Karppinen, M. *et al.* Oxygen nonstoichiometry in $YBaCo_4O_{7+\delta}$: large low-temperature oxygen absorption/desorption capability. *Chemistry of materials* **18**, 490-494 (2006).
24 Kendrick, E., Kendrick, J., Knight, K. S., Islam, M. S. & Slater, P. R. Cooperative mechanisms of fast-ion conduction in gallium-based oxides with tetrahedral moieties. *Nature materials* **6**, 871-875 (2007).
25 Kuang, X. *et al.* Interstitial oxide ion conductivity in the layered tetrahedral network melilite structure. *Nature materials* **7**, 498-504 (2008).
26 Hong, W. T., Welsch, R. E. & Shao-Horn, Y. Descriptors of oxygen-evolution activity for oxides: a statistical evaluation. *The Journal of Physical Chemistry C* **120**, 78-86 (2016).
27 Mefford, J. T. *et al.* Water electrolysis on $La_{1-x}Sr_xCoO_{3-\delta}$ perovskite electrocatalysts. *Nature communications* **7**, 1-11 (2016).
28 Trotochaud, L., Ranney, J. K., Williams, K. N. & Boettcher, S. W. Solution-Cast Metal Oxide Thin Film Electrocatalysts for Oxygen Evolution. *Journal of the American Chemical Society* **134**, 17253-17261, (2012).
29 Bockris, J. O. M. & Otagawa, T. The Electrocatalysis of Oxygen Evolution on Perovskites. *Journal of The Electrochemical Society* **131**, 290-302, (1984).
30 Calle-Vallejo, F. *et al.* Number of outer electrons as descriptor for adsorption processes on transition metals and their oxides. *Chemical Science* **4**, 1245-1249 (2013).
31 Man, I. C. *et al.* Universality in oxygen evolution electrocatalysis on oxide surfaces. *ChemCatChem* **3**, 1159-1165 (2011).
32 Song, J. *et al.* A review on fundamentals for designing oxygen evolution electrocatalysts. *Chemical Society Reviews* **49**, 2196-2214 (2020).
33 Hong, W. T. *et al.* Charge-transfer-energy-dependent oxygen evolution reaction mechanisms for perovskite oxides. *Energy & Environmental Science* **10**, 2190-2200 (2017).
34 Hwang, J. *et al.* Perovskites in catalysis and electrocatalysis. *Science* **358**, 751-756 (2017).
35 Grimaud, A. *et al.* Activating lattice oxygen redox reactions in metal oxides to catalyse oxygen evolution. *Nature chemistry* **9**, 457-465 (2017).
36 Sun, S. *et al.* Shifting oxygen charge towards octahedral metal: a way to promote water oxidation on cobalt spinel oxides. *Angewandte Chemie* **131**, 6103-6108 (2019).
37 Li, H. *et al.* Degree of geometric tilting determines the activity of FeO6 octahedra for water oxidation. *Chemistry of Materials* **30**, 4313-4320 (2018).
38 Kobussen, A. & Broers, G. The oxygen evolution on $La_{0.5}Ba_{0.5}CoO_3$: theoretical impedance behaviour for a multi-step mechanism involving two adsorbates. *Journal of Electroanalytical Chemistry and Interfacial Electrochemistry* **126**, 221-240 (1981).

39 Subbaraman, R. *et al.* Trends in activity for the water electrolyser reactions on 3d M (Ni, Co, Fe, Mn) hydr (oxy) oxide catalysts. *Nature materials* **11**, 550-557 (2012).
40 Lu, Z. *et al.* Electrochemical tuning of layered lithium transition metal oxides for improvement of oxygen evolution reaction. *Nature Communications* **5**, 4345, (2014).
41 Kanan, M. W. & Nocera, D. G. In Situ Formation of an Oxygen-Evolving Catalyst in Neutral Water Containing Phosphate and Co^{2+}. *Science* **321**, 1072-1075, (2008).
42 Risch, M. *et al.* Cobalt−Oxo Core of a Water-Oxidizing Catalyst Film. *Journal of the American Chemical Society* **131**, 6936-6937, (2009).
43 Bajdich, M., García-Mota, M., Vojvodic, A., Nørskov, J. K. & Bell, A. T. Theoretical Investigation of the Activity of Cobalt Oxides for the Electrochemical Oxidation of Water. *Journal of the American Chemical Society* **135**, 13521-13530, (2013).
44 Zhang, M., de Respinis, M. & Frei, H. Time-resolved observations of water oxidation intermediates on a cobalt oxide nanoparticle catalyst. *Nature Chemistry* **6**, 362-367, (2014).
45 Plaisance, C. P. & van Santen, R. A. Structure Sensitivity of the Oxygen Evolution Reaction Catalyzed by Cobalt(II,III) Oxide. *Journal of the American Chemical Society* **137**, 14660-14672, (2015).
46 Fernando, A. & Aikens, C. M. Reaction Pathways for Water Oxidation to Molecular Oxygen Mediated by Model Cobalt Oxide Dimer and Cubane Catalysts. *The Journal of Physical Chemistry C* **119**, 11072-11085, (2015).
47 Mattioli, G., Giannozzi, P., Amore Bonapasta, A. & Guidoni, L. Reaction Pathways for Oxygen Evolution Promoted by Cobalt Catalyst. *Journal of the American Chemical Society* **135**, 15353-15363, (2013).
48 Huang, Z.-F. *et al.* Chemical and structural origin of lattice oxygen oxidation in Co-Zn oxyhydroxide oxygen evolution electrocatalysts. *Nature Energy* **4**, 329-338, (2019).
49 Wang, L.-P. & Van Voorhis, T. Direct-Coupling O2 Bond Forming a Pathway in Cobalt Oxide Water Oxidation Catalysts. *The Journal of Physical Chemistry Letters* **2**, 2200-2204, doi:10.1021/jz201021n (2011).
50 Jun, K. *et al.* Lithium superionic conductors with corner-sharing frameworks. *Nature Materials*, 1-8 (2022).
51 Fei, H. *et al.* General synthesis and definitive structural identification of MN4C4 single-atom catalysts with tunable electrocatalytic activities. *Nature Catalysis* **1**, 63-72 (2018).
52 Gao, Z. W. *et al.* Engineering NiO/NiFe LDH intersection to bypass scaling relationship for oxygen evolution reaction via dynamic tridimensional adsorption of intermediates. *Advanced materials* **31**, 1804769 (2019).
53 Doyle, A. D., Montoya, J. H. & Vojvodic, A. Improving oxygen electrochemistry through nanoscopic confinement. *ChemCatChem* **7**, 738-742 (2015).
54 Bergmann, A. *et al.* Reversible amorphization and the catalytically active state of crystalline Co_3O_4 during oxygen evolution. *Nature Communications* **6**, 8625, (2015).
55 Bergmann, A. *et al.* Unified structural motifs of the catalytically active state of Co(oxyhydr)oxides during the electrochemical oxygen evolution reaction. *Nature Catalysis* **1**, 711-719, (2018).
56 Okamura, M. *et al.* A pentanuclear iron catalyst designed for water oxidation. *Nature* **530**, 465-468, (2016).
57 Lee, S. W. *et al.* The Nature of Lithium Battery Materials under Oxygen Evolution Reaction Conditions. *Journal of the American Chemical Society* **134**, 16959-16962, (2012).

58 Chmaissem, O., Zheng, H., Huq, A., Stephens, P. W. & Mitchell, J. F. Formation of Co^{3+} octahedra and tetrahedra in $YBaCo_4O_{8.1}$. *Journal of Solid State Chemistry* **181**, 664-672, (2008).
